# Transcriptome analysis of sugarcane reveals differential switching of major defense signaling pathways in response to *Sporisorium scitamineum* isolates with varying virulent attributes

**DOI:** 10.3389/fpls.2022.969826

**Published:** 2022-10-17

**Authors:** V.N. Agisha, N.M.R. Ashwin, R.T. Vinodhini, Kumaravel Nalayeni, Amalraj Ramesh Sundar, Palaniyandi Malathi, Rasappa Viswanathan

**Affiliations:** Division of Crop Protection, Indian Council of Agricultural Research (ICAR)-Sugarcane Breeding Institute, Coimbatore, India

**Keywords:** smut, sugarcane, plant defense, plant-pathogen interactions, RNA sequencing

## Abstract

Sugarcane smut caused by the basidiomycetous fungus *Sporisorium scitamineum* is one of the most devastating diseases that affect sugarcane production, globally. At present, the most practical and effective management strategy for the disease is the cultivation of resistant cultivars. In this connection, a detailed understanding of the host’s defense mechanism in response to smut isolates with varying degrees of virulence at the molecular level would facilitate the development of reliable and durable smut-resistant sugarcane varieties. Hence, in this study, a comparative whole transcriptome analysis was performed employing Illumina RNA-seq in the smut susceptible cultivar Co 97009 inoculated with two distinct *S. scitamineum* isolates, Ss97009 (high-virulent) and SsV89101 (low-virulent) during the early phases of infection (2 dpi and 5 dpi) and at the phase of sporogenesis (whip emergence) (60 dpi). Though the differential gene expression profiling identified significant transcriptional changes during the early phase of infection in response to both the isolates, the number of differentially expressed genes (DEGs) were more abundant at 60 dpi during interaction with the high virulent isolate Ss97009, as compared to the low virulent isolate SsV89101. Functional analysis of these DEGs revealed that a majority of them were associated with hormone signaling and the synthesis of defense-related metabolites, suggesting a complex network of defense mechanisms is being operated in response to specific isolates of the smut pathogen. For instance, up-regulation of hormone-related genes, transcription factors, and flavonoid biosynthesis pathway genes was observed in response to both the isolates in the early phase of interaction. In comparison to early phases of infection, only a few pathogenesis-related proteins were up-regulated at 60 dpi in response to Ss97009, which might have rendered the host susceptible to infection. Strikingly, few other carbohydrate metabolism-associated genes like invertases were up-regulated in Ss97009 inoculated plants during the whip emergence stage, representing a shift from sucrose storage to smut symptoms. Altogether, this study established the major switching of defense signaling pathways in response to *S. scitamineum* isolates with different virulence attributes and provided novel insights into the molecular mechanisms of sugarcane-smut interaction.

## Introduction

Sugarcane (*Saccharum officinarum*) is one of the most important crops cultivated worldwide for sugar and biofuel production (de Souza et al., 2014; Manochio et al., 2017). India is the second major country producing 397.65 million tonnes of sugarcane from an area of 4.851 million ha in 2020-21 according to the Ministry of Agriculture and Farmers’ Welfare. Nonetheless, sugarcane production is affected by many biotic and abiotic stresses. Among the diseases, sugarcane smut caused by the biotrophic fungus *Sporisorium scitamineum* remains to be the major disease affecting global sugarcane production (Sundar et al., 2012). The main characteristic symptom of sugarcane smut is the modification of the infected meristem into a black whip-like structure harboring billions of teliospores. In addition, infected sugarcane also exhibits profuse tillering with spindle shoots and poor cane formation. Smut is disseminated through infected seed material and by wind- or rain-borne teliospores (Sundar et al., 2012). On the surface of young buds, the germinating teliospores undergo meiosis to produce haploid sporidia of opposite mating types (*MAT-1* and *MAT-2*). Mating compatible haploid sporidia fuse together to develop into pathogenic dikaryotic mycelia, which are capable of infecting the host plant (Agisha et al., 2022). The infective mycelia penetrate through the bud surface and systemically colonize the apical meristem resulting in host infection (Izadi and Moosawi-Jorf, 2007).

The disease severity of sugarcane smut varies greatly, ranging from 10-80%, depending on the climatic conditions, the prevalence of pathogen races, and the resistance of the sugarcane varieties under cultivation in a given area (Monteiro-Vitorello et al., 2018; Nalayeni et al., 2021). Until now, the most effective management strategy for sugarcane smut is the use of resistant cultivars (Sundar et al., 2015; Sundar et al., 2018). In this regard, knowledge of the differential responses of sugarcane to pathogen infection is of pivotal importance, and the identification of differentially expressed transcripts has greater potential to develop genetic markers for disease resistance in sugarcane. Previously, differential expression study of sugarcane genes during *S. scitamineum* interaction was carried out by many research groups using various techniques viz., suppression-subtractive hybridization (SSH) and complementary DNA - amplified fragment length polymorphisms (cDNA-AFLP) (Lao et al., 2008; Huang et al., 2018).

RNA-seq utilizing high-throughput Next-Generation Sequencing (NGS) delivers an unbiased view of the transcriptome and also provides information on minor variations in gene expression. So far, many transcriptomic studies have been adopted to elucidate plant-fungal interactions in different crops (Naidoo et al., 2018). Owing to the global prevalence of sugarcane smut, new NGS platforms like Illumina sequencing technology were employed for comparative expression profiling in sugarcane genotypes during interaction with the smut pathogen. Que et al. (2014) analyzed the transcriptome of sugarcane infected with *S. scitamineum* at 24, 48, and 120 h, using an *S. scitamineum*-resistant and -susceptible genotype (Yacheng05-179 and ‘‘ROC’’22). Subsequently, the transcriptional changes leading to disease symptoms in a smut intermediate-resistant sugarcane genotype were investigated (Schaker et al., 2016). Likewise, the implication of internal and external resistance mechanisms in sugarcane during *S. scitamineum* interaction was studied in a resistant cultivar (CP74-2005) using RNA-seq (McNeil et al., 2018).

In India, smut is becoming a serious threat to sugarcane cultivation in both tropical and subtropical regions and is reported to cause yield loss of up to 50% (Viswanathan and Rao, 2011). Meanwhile, variability studies on the prevalence of *S. scitamineum* genotypes worldwide revealed that the genetic diversity among the Asian genotypes is more and extraordinarily diverse from American and African lineage (Raboin et al., 2007). Especially, the diversity was found to be more among Indian and Chinese pathotypes (Braithwhite et al., 2004; Shen et al., 2012; Barnabas et al., 2018). An assessment of fifty isolates of sugarcane smut pathogen representing seven major sugarcane growing regions in India revealed considerable genetic diversity and pathogenic variability among them, and clear discrimination of these isolates was evident based on the differential responses of a set of sugarcane varieties (Barnabas et al., 2018). For instance, many cultivars that were regarded as resistant to smut elsewhere (Gillaspie et al., 1983; Grisham, 2001; Schenck, 2003; Nzioki et al., 2010; Luzaran et al., 2012), exhibit susceptibility to Indian isolates. Given the scenario, hitherto, the application of NGS technology to decipher the host responses in response to different smut isolates from India with varying degrees of virulence was not explored.

In the present study, a comparative whole transcriptome of smut susceptible cultivar Co 97009 during interaction with a high- and a low-virulent *S. scitamineum* isolate, Ss97009 and SsV89101, respectively was profiled at 2, 5, and 60 dpi (days post-inoculation), which revealed the differential switching of defense-signaling pathways in sugarcane in response to *S. scitamineum* isolates with different virulence behaviors. Hence, this study would enhance the understanding of complex molecular mechanisms in sugarcane, which would eventually help in developing sugarcane cultivars with durable smut resistance either by molecular breeding or by transgenic approaches.

## Materials and methods

### Fungal isolates and plant material

Teliospores of a high-virulent *S. scitamineum* isolate, Ss97009, and a low-virulent isolate, SsV89101, were collected from the smut susceptible sugarcane cultivars, Co 97009 and CoV 89101, respectively, from the experimental farm of ICAR-SBI, Coimbatore, India, and used in this study. Previously, the virulence of these isolates was evaluated by relative virulence testing on a smut susceptible variety and confirmed by differential host experiments (Barnabas et al., 2018; Nalayeni et al., 2021). In the present study, single bud sets of 7-month-old Co 97009, a smut susceptible variety was used for plant inoculation experiments.

### Experimental design

Pre-germinated buds of the sugarcane cv. Co 97009 were inoculated with teliospores (4 x 10^6^ spores/mL) of *S. scitamineum* isolates Ss97009 and SsV89101 by bud pasting method (Co et al., 2008), planted, and maintained under glasshouse conditions at 28°C - 30°C with 60-80% humidity. At least twenty plants were inoculated per treatment and an equal number of uninoculated plants served as control. The inoculated plants were monitored regularly for the emergence of smut whips. Meristem samples comprising three biological replicates were collected each from inoculated and mock-inoculated control plants at 2 dpi, 5 dpi, and 60 dpi and used separately for scanning electron microscopy (SEM) and transcriptome analyses (**Supplementary Figure 1A**). Additionally, quantitative real-time PCR was used to estimate the pathogen biomass of plants inoculated with teliospores of Ss97009 and SsV89101 using the primers, qbE-F1 and qbE-R1, targeting *bE* mating type genes (Agisha et al., 2022).

### Histological observation of *in planta* developmental stages by scanning electron microscopy

To confirm fungal colonization, pathogen inoculated meristem samples collected at different time intervals viz., 2 dpi, 5 dpi, and 60 dpi were prepared for observation by scanning electron microscopy (Waikhom et al., 2014). The meristem samples were sectioned and fixed overnight in 2.5% glutaraldehyde in 0.1 M phosphate buffer (pH-7.4) at 4°C. After fixation, the samples were washed in phosphate buffer and dehydrated in 30% ethanol. The sections were then dried on carbon tape placed on an aluminium stub, coated with gold-palladium using a sputter coater (Emitech SC7620, UK), and observed under SEM (FEI Quanta 250, USA).

### RNA extraction, library construction, and Illumina sequencing

Total RNA from the meristem tissues of inoculated (Ss97009 and SsV89101) and mock-inoculated control plants at 2 dpi, 5 dpi, and 60 dpi (three biological replications per sample) were extracted using the RNeasy Plant Mini kit (Qiagen, Germany) with on-column digestion using DNase enzyme (Qiagen, Germany) according to manufacturer’s instructions. RNA quality, integrity, and concentrations were assessed by agarose gel electrophoresis, NanoDrop (ThermoScientific, USA), and Qubit (Invitrogen, USA). The quality of the samples was further verified by determining the RNA Integrity Number (RIN) using Agilent 2100 Bioanalyzer (Agilent Technologies, USA). High-quality RNA samples with RIN value >6.0 were used for the construction of cDNA libraries using the TruSeq RNA Sample Prep Kits (Illumina, USA) as described in the manufacturer’s instructions. A total of 27 libraries (3 libraries/sample) were sequenced on HiSeq 4000 (Illumina, USA) to generate paired-end reads (2x150 bp) of about 5-10 GB quality reads/library.

### Data pre-processing and *de novo* assembly of sugarcane reads

The quality of raw reads was evaluated by FastQC software (https://www.bioinformatics.babraham.ac.uk/projects/fastqc/) with the set parameters of base quality score distribution, sequence quality score distribution, and average base content per read. The adaptor sequences and the reads with an average Phred quality score of less than 20 were trimmed using Adapter Removal-v2 (version 2.2.0), while also removing reads shorter than 30 bp. Further, the contaminant rRNA sequences were removed by aligning the reads with the silva database (https://www.arb-silva.de/) using Bowtie2 (version 2.2.9). Fungal reads were removed by mapping them to the *S. scitamineum* reference genome (ftp://ftp.ncbi.nlm.nih.gov/genomes/all/GCA/900/002/365/GCA_900002365.1_ASM90000236v1/GCA_900002365.1_ASM90000236v1_genomic.fna.gz). The unaligned reads representing the host (i.e., sugarcane) were normalized and carried forward for *de novo* transcriptome assembly using Trinity (version 2.9.1). The assembled transcripts were then clustered into unigenes using the same trinity pipeline and these unigenes were annotated against the Uniprot database using BLAST with the taxonomy filter, Viridiplantae, and E-value of 1.0E-3. Differential gene expression analysis was performed for chosen pairs of samples followed by functional annotation. The workflow of the data analysis approach is summarized in **Supplementary Figure 1B**.

### Differential gene expression analysis

For Differential Gene Expression (DGE) analysis, transcript abundance and counts of the assembled transcripts were obtained using Kallisto (version 0.46.0). To compare the differential expression between the two isolates Ss97009 and SsV89101 at different time intervals, six pairwise comparisons viz., 2 dpi Ss97009 vs 2 dpi control, 2 dpi SsV89101 vs 2 dpi control, 5 dpi Ss97009 vs 5 dpi control, 5 dpi SsV89101 vs 5 dpi control, 60 dpi Ss97009 vs 60 dpi control and 60 dpi SsV89101 vs 60 dpi control were analyzed. DGE analysis was carried out based on the software package edgeR in OmicsBox version 1.4.11 (Robinson et al., 2010). The effective library size was normalized using the Trimmed Mean of M-values (TMM) normalization method, and low count genes were filtered by selecting genes with at least 1 count-per-million (CPM). Quasi-likelihood F-test was employed to determine differentially expressed genes. The p-values generated from the edgeR analysis were adjusted for false discovery rates (FDR) across the multiple tests by Benjamini-Hochberg method. The FDR value < 0.05 and log fold change of 2 (-2≥ LogFC ≥2) were used as the thresholds to judge the significance of differences in gene expression for each time point comparison. Venn diagrams were plotted using the jvenn tool (http://jvenn.toulouse.inra.fr/app/index.html) to find out the differential co-expression in plants inoculated with Ss97009 and SsV89101 isolates.

### Functional annotation

To understand the molecular function of the DEGs, OmicsBox version 1.4.11 and Blast2GO 5 PRO package were used with the default parameters. The FASTA sequences of the DEGs were searched against NCBI non-redundant (Nr) protein database using BLASTx with the taxonomy filter, Viridiplantae, and E-value of 1.0E-3. The BLAST result accessions and BLAST hit IDs were mapped to retrieve their Gene Ontology (GO) terms by searching against different databases viz., GO database, UniProt, Swiss-Prot, TrEMBL, RefSeq, GenPept, and PDB. InterProScan annotation was done by searching against the public repository EMBL-EBI to retrieve the domain/motif information of the sequences. Also, enzyme code annotations were retrieved for the sequences with GO annotations. Subsequently, linking all the differentially expressed genes to the biological pathways was performed using the KEGG (Kyoto Encyclopedia of Genes and Genomes) database. The Cluster of Orthologous Groups (COG) annotation was done using EggNOG (Evolutionary genealogy of genes: Non-supervised Orthologous Groups) based orthology assignments.

### Validation of DEGs by quantitative PCR

Quantitative real-time PCR analysis was done to validate candidate DEGs obtained from Illumina RNA-seq. Primers were designed using NCBI primer-BLAST (https://www.ncbi.nlm.nih.gov/tools/primer-blast/index.cgi?INPUT_SEQUENCE=%20EU563945.2&LINK_LOC=nuccore) and the quality of the primers were verified using Oligo Calc: Oligonucleotide Properties Calculator (http://biotools.nubic.northwestern.edu/OligoCalc.html) and Sigma-Aldrich OligoEvaluator (http://www.oligoevaluator.com/LoginServlet). The list of candidate genes and primers used for qPCR validation of RNA-seq analysis are given in **Supplementary Table 1**. qPCR was performed with the same set of RNA samples with triplicates that were used for transcriptome analysis and cDNA was synthesized by reverse transcription using RevertAid H Minus reverse transcriptase (Thermo Scientific, USA). The reactions were carried out in a StepOnePlus Real-time PCR system (Applied Biosystems, USA) with the SensiFAST SYBR Hi-ROX Kit (Bioline, USA). The total reaction volume was 20 µl containing 50 ng of template, 10 µl of SensiFAST SYBR Hi-ROX Master Mix, and 0.05 µM of each primer. The conditions used for amplification were as follows: initial denaturation at 95°C for 10 min followed by 40 cycles of denaturation at 95°C for 15 s and annealing at 60°C for 1 min. For quantification of relative gene expression levels, the 2^-ΔΔCt^ algorithm was applied (Livak and Schmittgen, 2001). The sugarcane housekeeping gene encoding elongation factor 1α (eEF1α), was used as the reference gene to normalize the expression of the target genes (Ashwin et al., 2020a). The significance of the relative expression between the combinations was determined at p ≤ 0.05 based on the post-hoc Tukey’s test by employing SPSS statistical software version 21 (IBM, USA).

## Results

### Disease development and detection of *S. scitamineum* infection at distinct *in planta* colonization stages in sugarcane by scanning electron microscopy

The virulence pattern of the *S. scitamineum* isolates Ss97009 and SsV89101 was re-established by phenotyping the magnitude of symptom expression in the smut susceptible sugarcane cv. Co 97009. The *S. scitamineum* isolate Ss97009 caused a total of 9 whips at 60 dpi, confirming its high virulence, while no whips emerged with the low-virulent isolate SsV89101 (**Supplementary Figure 2**). To investigate the dynamic transcript changes in sugarcane in response to these high- and low-virulent isolates, three successive stages during disease progression, i.e., meristem samples collected at 2 dpi, 5 dpi, and 60 dpi were subjected to transcriptome sequencing. Meanwhile, the differences in the infection process of the two isolates were analyzed through scanning electron microscopy (SEM), to optimize the time intervals of sampling for RNA-seq analysis. Extensive colonization of the external surface of the buds with germinating teliospores and mycelia was observed with Ss97009 and SsV89101 at 2 dpi. Over time, bulbous structures attempting penetration on the internal surface and intracellular colonization were detected at 5 dpi with both the isolates. During the later biotrophic phase (60 dpi), Ss97009 showed extensive sporogenesis in meristem with fragmented hyphae and teliospores, whereas considerably less colonization was observed with SsV89101, reinforcing its lower virulence than Ss97009. An illustrative image displaying the different developmental stages of *S. scitamineum* on interaction with sugarcane is depicted in **Supplementary Figure 3**. Quantitative real-time PCR confirmed the differences in pathogen biomass in sugarcane during colonization with the isolates Ss97009 and SsV89101 (**Supplementary Figure 4**). Owing to the presence of inoculum on the bud surface, a similar amount of fungal DNA (~4 ng) was detected at 2 dpi in plants challenge inoculated with both isolates. At 5 dpi, the high-virulent isolate Ss97009 had a substantially higher amount of fungal DNA (~2 ng) than SsV89101, which had 0.5 ng, indicating that the high-virulent isolate had better penetration of infective dikaryotic mycelia. Notably, the pathogen biomass of Ss97009 at 60 dpi (~22 ng) was found to be higher than that of SsV89101 (~0.1 ng).

### Transcriptome sequencing and *de novo* transcriptome assembly

**Figure 1:**
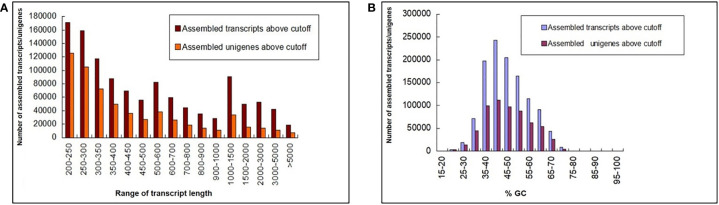
Length and GC content of the assembled transcripts/unigenes. **(A)** Bar chart depicting the length distribution and **(B)** GC summary of the assembled transcripts/unigenes. The y-axis represents the number of assembled transcripts/unigenes, the x-axis (left) represents the range of transcript length, and the x-axis (right) represents the %GC.

A total of 653 million paired-end (PE) raw reads (~196 GB) of 150 bp were generated from 27 libraries of 9 *in planta* samples (three biological replicates each from 2 dpi, 5 dpi, and 60 dpi of control, Ss97009, and SsV89101 inoculated samples), so as to understand the molecular mechanisms of sugarcane in response to infection with Ss97009 and SsV89101. Totally, 238 million reads (36.4%) were obtained for 2 dpi samples, 190 million reads (29.1%) for 5 dpi samples, and 225 million (34.5%) for 60 dpi samples. An average of 95.82% of total reads passed ≥ 30 Phred score. Details of the number of raw reads generated (paired-end), GC%, and Q30 scores for each sample are listed in **Supplementary Table 2**. Alignment with the silva database resulted in the removal of 23% of raw reads containing contaminant rRNA sequences. Further, fungal reads were removed after mapping them to the *S. scitamineum* whole genome (Dutheil et al., 2016), resulting in the removal of approximately 3.3% of the reads. The pre-processed host reads were then carried forward for assembly using Trinity software (**Supplementary Table 3**). The total number of unigenes identified for this *de novo* transcriptome assembly was 6,06,157 with an N50 of 585 bp, while the total transcripts (including isoforms) were 11,65,148 with an N50 length of 689 bp. The length distribution of the transcriptome was analyzed and all the assembled transcripts were ≥ 200. The mean GC% of the total transcripts and unigenes were 46.49 and 46.66, respectively. Length and GC content of the assembled transcripts/unigenes are summarized in **Figure 1**, and the *de novo* transcriptome assembly statistics are summarized in **Supplementary Table 4**. A total of 3,06,787 unigenes were annotated against the Uniprot database. The species distribution revealed that 45,956 of the unigenes showed significant homology with *Sorghum bicolor*, 31,767 showed significant homology with *Zea mays*, and 11,295 showed significant homology with *Oryza sativa*.

### Differential gene expression profiling of sugarcane inoculated with *S. scitamineum* isolates

Differential gene expression analysis identified the DEGs in samples inoculated with the *S. scitamineum* isolates Ss97009 and SsV89101 during distinct stages of disease progression viz., 2 dpi, 5 dpi and 60 dpi (**Supplementary File 1**). The numbers of up-regulated and down-regulated DEGs at these time intervals in both the isolates were summarized in **Table 1**. A rise in the number of DEGs was demonstrated with both the isolates during disease progression from 2 dpi to 5 dpi. Remarkably, DEGs were more abundant in the high-virulent as compared to the low-virulent isolate during the later stage of infection (60 dpi). In general, the number of up-regulated genes was higher than the down-regulated genes at all time points except at 60 dpi of the SsV89101 inoculated sample. Among the distinct stages of disease progression, the number of DEGs was higher in the early stages (2 dpi and 5 dpi) than in the later stage in response to both the isolates. Notably, Ss97009 inoculated plants had a greater number of DEGs than SsV89101 inoculated plants at 2 dpi and 60 dpi, while the number of DEGs in response to both Ss97009 and SsV89101 was nearly the same at 5 dpi.

**Table 1:** Differentially expressed genes between pairwise sample combinations.

SI.No	Samples	Up-regulated genes	Down-regulated genes	Total number of DEGs
1	2 dpi Ss97009 *vs* 2 dpi control	888	166	1,054
2	2 dpi SsV89101 *vs* 2 dpi control	364	82	446
3	5 dpi Ss97009 *vs* 5 dpi control	788	650	1,438
4	5 dpi SsV89101 *vs* 5 dpi control	1,042	523	1,565
5	60 dpi Ss97009 *vs* 60 dpi control	550	189	739
6	60 dpi SsV89101 *vs* 60 dpi control	79	86	165

Unigenes with a false discovery rate (FDR) no greater than 0.05 and log2 fold-change ≥2 and ≤-2 were considered to be differentially expressed genes (DEGs).

The differential co-expression in plants inoculated with Ss97009 and SsV89101 isolates at the same time point from three groups: (1) DEGs (2dpi Ss97009 vs 2dpi control) vs (2dpi SsV89101 vs 2dpi control), (2) DEGs (5dpi Ss97009 vs 5dpi control) vs (5dpi SsV89101 vs 5dpi control), and (3) DEGs (60dpi Ss97009 vs 60dpi control) vs (60dpi SsV89101 vs 60dpi control) were identified (**Supplementary Figure 5A**; **Supplementary File 2**). This paired comparison showed a strong overlap in the DEGs during the early stages (2 dpi and 5 dpi) compared to the later stage (60 dpi). Besides, differential co-expression among the Ss97009 and SsV89101 inoculated plants at different time points: (1) (2dpi Ss97009 vs 2dpi control) vs (5dpi Ss97009 vs 5dpi control) vs (60dpi Ss97009 vs 60dpi control), and (2) (2dpi SsV89101 vs 2dpi control) vs (5dpi SsV89101 vs 5dpi control) vs (60dpi SsV89101 vs 60dpi control) were also determined (**Supplementary Figure 5B**; **Supplementary File 2**). These findings revealed that both Ss97009 and SsV89101 had many unique DEGs at all time points. Remarkably, the number of specific DEGs at 60 dpi was found to be more than four times higher with the high-virulent as compared to the low-virulent. Regarding common DEGs, more were found between 2 and 5 dpi than at 60 dpi for both isolates.

### Functional annotation of differentially expressed genes

**Figure 2:**
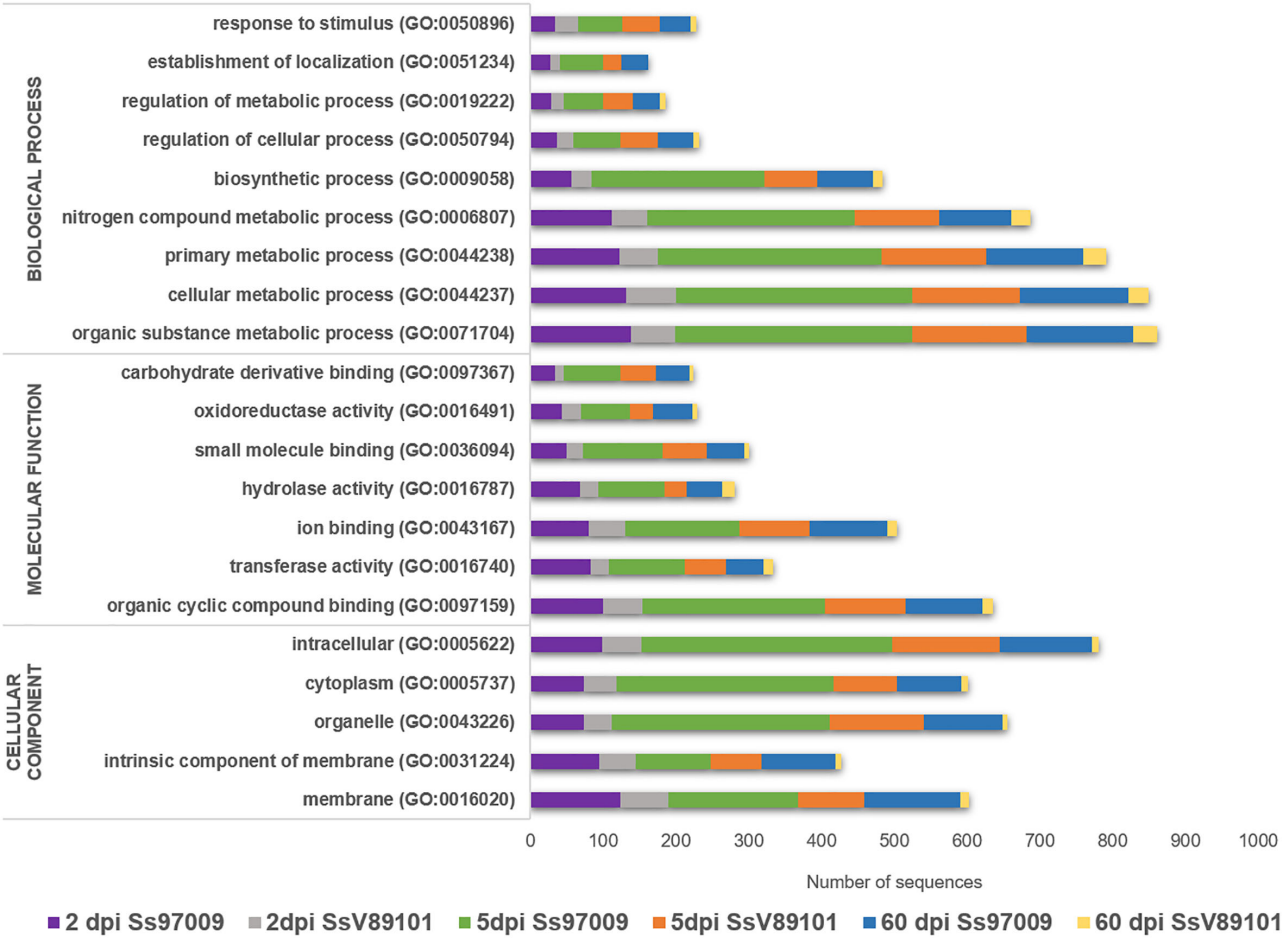
Gene ontology (GO) annotation of differentially expressed genes. The x-axis represents the number of sequences and the y-axis represents the GO terms under the main categories; biological process, molecular function, and cellular component.

Functional analysis through BLASTx, GO, InterProScan, Enzyme code, and COG annotations resulted in the identification of the biological functions of the DEGs. In Ss97009 inoculated plants, 67.4%, 57.0%, and 65.0% of the DEGs showed blast hits for 2 dpi, 5 dpi, and 60 dpi samples, respectively. Likewise, in the case of SsV89101 inoculated plants, 54.7%, 55.6% and 48.5% of the DEGs showed blast hits for 2 dpi, 5 dpi, and 60 dpi samples, respectively (**Supplementary File 3**). Further, DEGs under the main GO categories, biological process, molecular function, and cellular component were grouped. A total of 57.2%, 82.6%, and 81.3% of the DEGs in Ss97009 inoculated, and 77.8%, 85.0%, and 66.3% in SsV89101 inoculated plants at 2 dpi, 5 dpi, and 60 dpi, respectively, were annotated (**Figure 2**; **Supplementary File 4**). In Ss97009 and SsV89101 inoculated plants, the most represented GO terms under the molecular function category included organic cyclic compound binding, ion binding, and transferase activity irrespective of the time intervals. Under the biological process category, the GO terms were mostly related to cellular metabolic process, organic substance metabolic process, primary metabolic process, nitrogen compound metabolic process, and biosynthetic process, and the GO terms under cellular component were membrane, intracellular anatomical structure, organelle, and cytoplasm with both the isolates at all the time intervals. In all of these categories, Ss97009 stimulated the expression of a greater number of genes than SsV89101. Intriguingly, activation of defense associated categories viz., response to stress, establishment of localization, hydrolase activity, and oxidoreductase activity were also mostly linked to the high virulent isolate Ss97009. InterProScan annotation provided functional analysis of proteins by classifying them into families and predicting domains and important sites (**Supplementary File 5**). Major families included p-loop containing nucleoside triphosphate hydrolase (IPR027417), cytochrome P450 (IPR036396), DNA/RNA polymerase superfamily (IPR043502), etc. The domains viz., heme peroxidase (IPR002016) and glutathione S-transferase (IPR004045), protein kinase domain (IPR000719, IPR011009), etc. were identified with both the isolates, while homeobox domain (IPR001356) and multicopper oxidase (IPR011707, IPR011706) were the most represented in Ss97009 inoculated plants at 60 dpi. The most represented sites were cytochrome P450, conserved site (IPR017972), ubiquitin conserved site (IPR019954), and protein kinase, ATP binding site (IPR017441), etc., except in SsV89101 inoculated plants at 60 dpi. Enzymes representing the classes oxidoreductases, transferases, hydrolases, lyases, isomerases, and ligases were identified in the DEGs’ enzyme code annotations. Among these classes, oxidoreductases, transferases, and hydrolases represented the higher number of DEGs in both Ss97009 and SsV89101 inoculated plants. Remarkably, all of these classes exhibited a higher number of genes with Ss97009 than with SsV89101 during all the time frames (**Supplementary File 6**).

**Figure 3:**
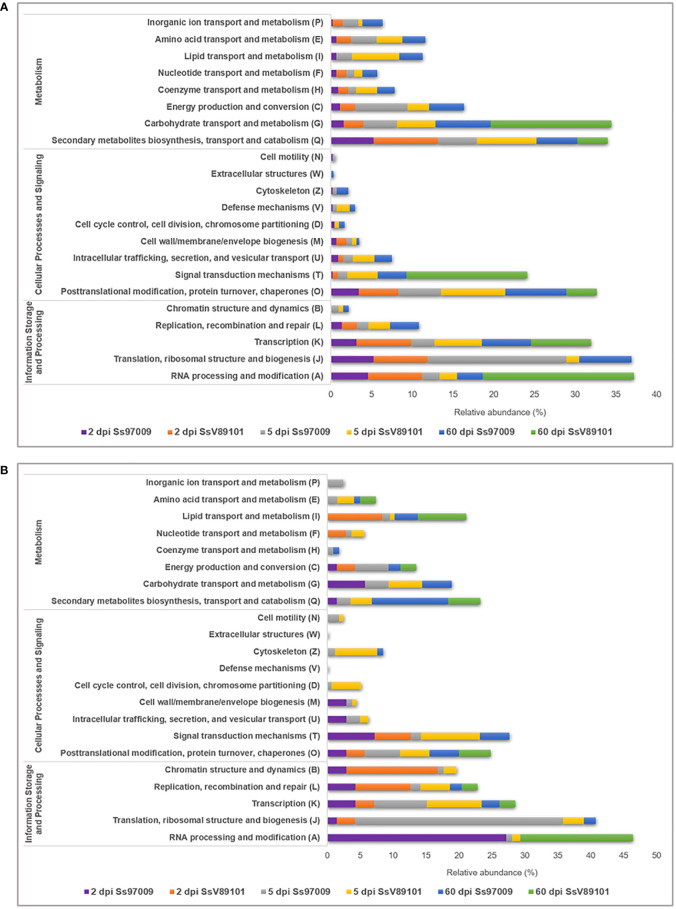
Functional distribution of Orthologous groups of proteins (COG) annotation of the differentially expressed genes in sugarcane inoculated with the *S. scitamineum* isolates Ss97009 and SsV89101. Distribution of COG categories in **(A)** up-regulated and **(B)** down-regulated DEGs. The x-axis represents the relative abundance (%) and the y-axis represents the COG categories.

COG annotation provided data on the DEGs under the COG categories viz., information storage and processing, metabolism, and cellular processes and signaling (**Supplementary File 7**). Major categories distributed among the DEGs included RNA processing and modification (A), translation, ribosomal structure and biogenesis (J), and transcription (K). (**Figure 3**). Notably, activation of these categories was more prominent in the early stages with both isolates than in the later stages, with Ss97009 having a greater number of DEGs than SsV89101. COG categories associated with defense responses such as signal transduction mechanisms (T), energy production and conversion (C), defense mechanisms (V), and inorganic ion transport and metabolism (P) were also differentially expressed. Interestingly, the number of DEGs was considerably larger with both isolates at 5 dpi, with Ss97009 demonstrating a higher number of DEGs than SsV89101. Other important categories included secondary metabolites biosynthesis, transport, and catabolism (Q), and carbohydrate transport and metabolism (G). The number of DEGs under these categories was substantially expressed in response to Ss97009, in contrast to SsV89101.

### Pathway distribution of differentially expressed genes

**Figure 4:**
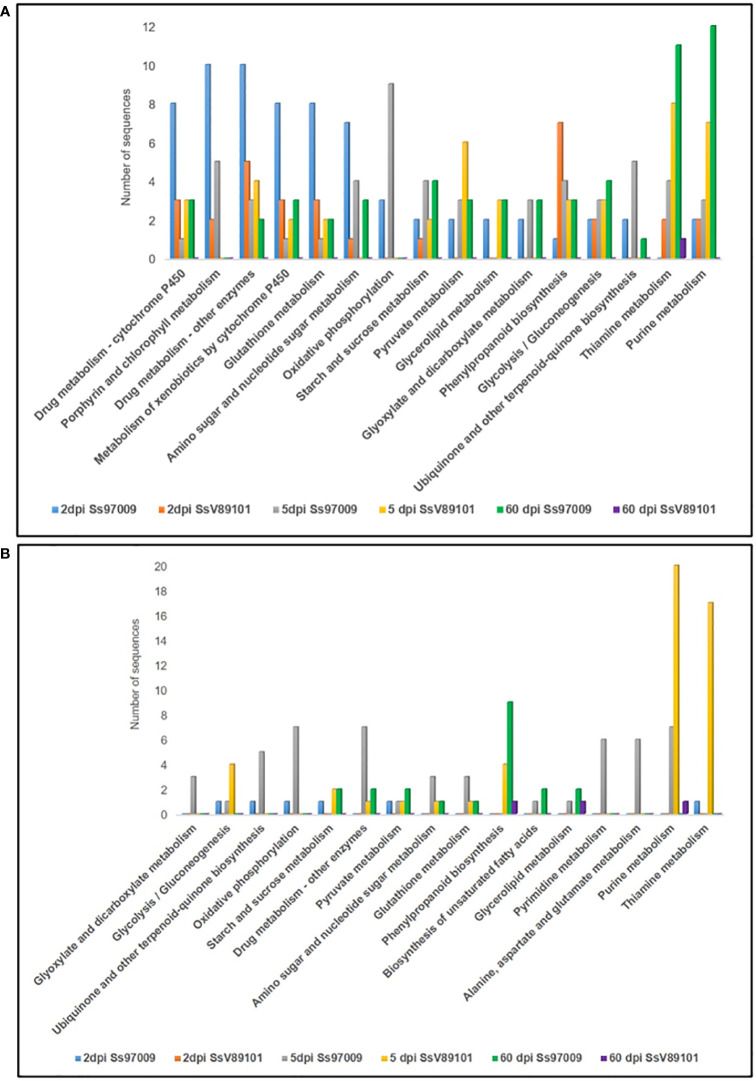
Kyoto Encyclopedia of Genes and Genomes (KEGG) pathway annotation of the differentially expressed genes in sugarcane inoculated with the *S. scitamineum* isolates Ss97009 and SsV89101. Distribution of KEGG pathways in **(A)** up-regulated and **(B)** down-regulated DEGs. The x-axis represents the number of genes and the y-axis represents the KEGG categories.

KEGG metabolic pathway analysis has depicted the pathways of the DEGs in sugarcane regulated upon inoculation with the *S. scitamineum* isolates Ss97009 and SsV81901 (**Figure 4**; **Supplementary File 8**). The most prominent pathways up-regulated at 2 dpi in Ss97009 inoculated plants included drug metabolism, porphyrin and chlorophyll metabolism, and glutathione metabolism, while in SsV89101 inoculated plants, phenylpropanoid biosynthesis, and drug metabolism were the most represented pathways. Among these, the phenylpropanoid biosynthesis pathway was outstandingly up-regulated with SsV89101 as compared to Ss97009 at 2 dpi. In response to Ss97009, amino sugar and nucleotide sugar metabolism, and oxidative phosphorylation were the most prominently up-regulated pathways at 5 dpi, while purine metabolism, pyrimidine metabolism, and alanine, aspartate and glutamate metabolism were found to be down-regulated at this time point. With the low-virulent isolate, thiamine metabolism, purine metabolism, and pyruvate metabolism were among the up-regulated pathways, whereas glycolysis/gluconeogenesis, purine metabolism, and thiamine metabolism were remarkably down-regulated at 5 dpi. Interestingly, the down-regulation of glycolysis/gluconeogenesis was higher in SsV89101 than in Ss97009 at 5 dpi. At the later biotrophic phase (60 dpi), genes involved in pathways viz., purine metabolism, thiamine metabolism, glycolysis/gluconeogenesis, and starch and sucrose metabolism were substantially up-regulated during the sporogenesis stage with Ss97009, while in SsV89101 inoculated asymptomatic plants, a few genes involved in terpenoid backbone biosynthesis, diterpenoid biosynthesis, and thiamine metabolism were observed to be up-regulated. Strikingly, metabolism-related pathways, as well as defense-related pathways, were found to be substantially regulated in the high-virulent as compared to SsV89101 at 60 dpi.

### Identification of differentially expressed defense-related genes

**Figure 5:**
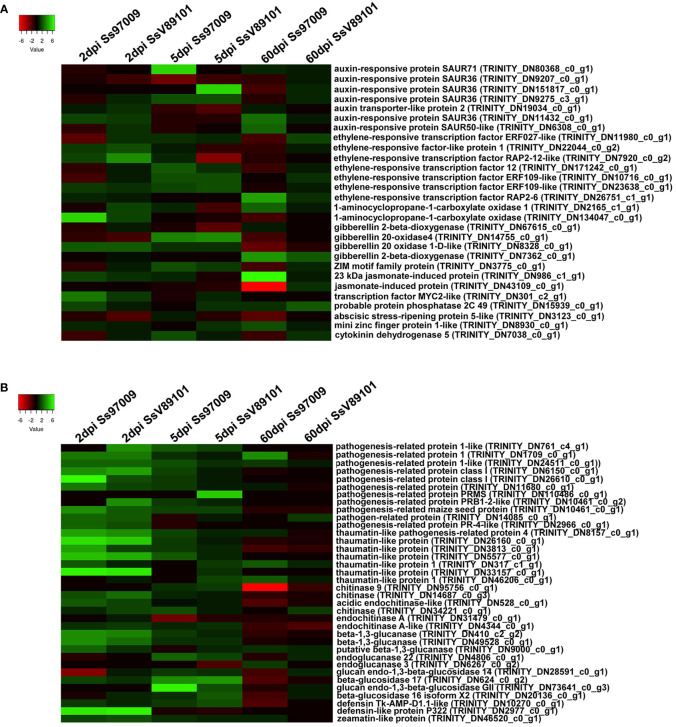
Heatmap of the differentially expressed genes (DEGs) in sugarcane cultivar Co 97009 inoculated with the *S. scitamineum* isolates Ss97009 and SsV89101. **(A)** DEGs related to hormone biosynthesis and **(B)** pathogenesis-related proteins with both the isolates at different time intervals (2 dpi, 5 dpi, and 60 dpi). Heatmaps were constructed using the log2 fold change values, and the genes in green and red represent up- and down-regulated genes respectively.

Changes in the expression profiles of defense-related genes viz., plant hormone-related genes, pathogenesis-related genes, transcription factors (TFs), genes involved in flavonoid biosynthesis pathway, and ROS-related genes were explored (**Supplementary File 9**). In our study, a total of 28 hormone-related genes including auxin, ethylene (ET), gibberellin (GA), and jasmonic acid (JA) which act as critical signals for regulating disease resistance in plants were differentially expressed under *S. scitamineum* stress (**Figure 5A**). At 2 dpi, 1-aminocyclopropane-1-carboxylate (ACC) oxidase involved in ET biosynthesis, gibberellin 20 oxidase 1-D-like (GA20ox1-D) involved in GA biosynthesis, and MYC2 transcription factor involved in JA signaling pathway were up-regulated in response to Ss97009. Concurrently, in SsV89101 inoculated plants, ethylene-responsive transcription factor (ERF) RAP2-12-like and ethylene-responsive factor-like protein 1, involved in the ET signaling pathway, and GA20ox1-D were induced at this time point, while no genes involved in JA were regulated. Over time, at 5 dpi, genes involved in ET and GA synthesis, as well as a ZIM motif family protein involved in the JA signaling pathway, were elevated in response to Ss97009. In contrast to 2 dpi, the auxin-responsive protein SAUR71, which is implicated in the auxin-activated signaling pathway, was up-regulated in response to Ss97009. Similarly, in SsV89101 infected plants, auxin-responsive protein SAUR36 was up-regulated in addition to ethylene-responsive transcription factor and gibberellin 20-oxidase 4, although no genes involved in the JA signaling pathway were up-regulated. Surprisingly, the maximum number of hormone-related genes were up-regulated in Ss97009 inoculated plants at 60 dpi, including ACC oxidase, 23 kDa jasmonate-induced protein, auxin-responsive proteins SAUR36 and SAUR50, ethylene-responsive transcription factor RAP2-6, and mini zinc finger protein 1. In contrast to early stages (2 and 5 dpi), gibberellin 20 oxidase 1-D involved in the production of active GAs was suppressed, and gibberellin 2-beta-dioxygenase involved in the deactivation of GAs was found elevated in response to Ss97009 at 60 dpi. Conversely, with the low-virulent SsV89101, only a few genes involved in hormone signaling pathways were found to be up-regulated at this time point.

Under *S. scitamineum* stress, a total of 36 pathogenesis-associated genes involved in response to pathogen infection were differentially expressed (**Figure 5B**). Remarkably, the highest number of up-regulated pathogenesis-related proteins was observed at 2 dpi, and beta 1,3 glucanase, chitinase, pathogenesis-related protein 1, thaumatin-like protein, and defensin-like protein P322 were up-regulated in both Ss97009 (19 Nos.) and SsV89101 (15 Nos.) inoculated plants. Over time, a total of 6 and 4 pathogenesis-related proteins were induced at 5 dpi in Ss97009 and SsV89101 inoculated plants, respectively. Intriguingly, at the whip emergence stage (60 dpi) in Ss97009 inoculated plants, pathogenesis-related genes were fewer (3 Nos.) compared to the early stages, and a chitinase was substantially suppressed. On the other hand, no pathogenesis-related genes were elevated in response to SsV89101 at 60 dpi.

**Figure 6:**
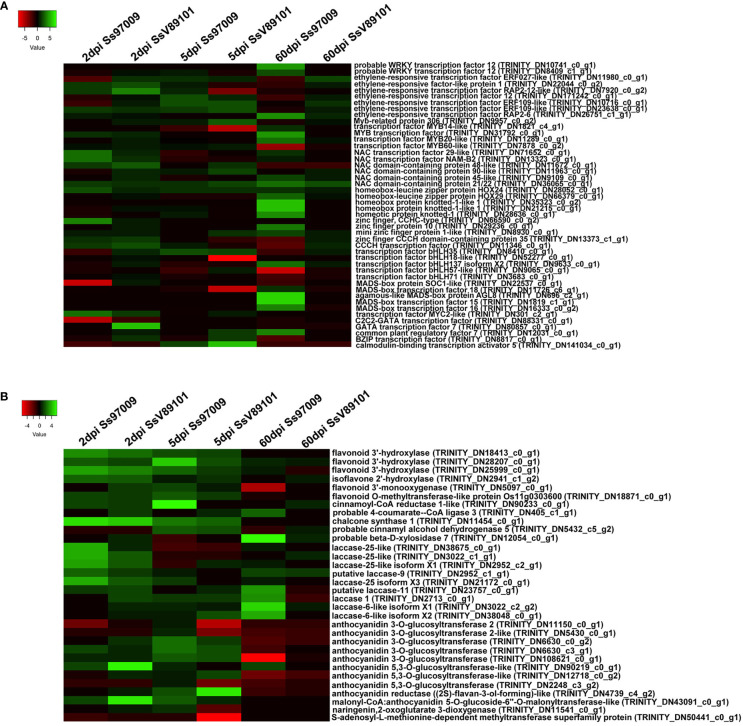
Heatmap of the differentially expressed genes (DEGs) in sugarcane cultivar Co 97009 inoculated with the *S. scitamineum* isolates Ss97009 and SsV89101. **(A)** DEGs related to transcription factors and **(B)** flavonoid biosynthesis pathway with both the isolates at different time intervals (2 dpi, 5 dpi, and 60 dpi). Heatmaps were constructed using the log2 fold change values, and the genes in green and red represent up- and down-regulated genes respectively.

Differential regulation of 48 defense-related transcription factors including NAC, ERF, bHLH, MYB, and WRKY were observed in the plants upon *S. scitamineum* stress (**Figure 6A**). During the early phases of infection (2 and 5 dpi), NAC transcription factor and ethylene-responsive factor, which are involved in defense responses were up-regulated in response to both Ss97009 and SsV89101. Meanwhile, up-regulation of bHLH35 transcription factors was identified only at 5 dpi with these two isolates. Though there was little difference in the expression of TFs between the two isolates in the early stages, Ss97009 inoculated plants showed a substantially higher expression at the whip emergence stage (60 dpi). At this time point, several defense-related transcription factors viz., ERF, NAC domain-containing proteins, zinc finger proteins, and bHLH transcription factor were significantly regulated in response to the high-virulent Ss97009. Besides, MYB transcription factor 20 and WRKY transcription factor 12 were also found to be induced exclusively at this stage. Notably, MADS-box transcription factors were *exclusively* up-regulated in the whip emerged plants inoculated with Ss97009. Conversely, in SsV89101 inoculated plants, no significant up-regulation of TFs was demonstrated except for an NAC domain-containing protein.

Genes involved in the flavonoid biosynthesis pathway, a class of polyphenolic secondary metabolites with important roles in many biological processes, were found to be differentially expressed in response to *S. scitamineum* (**Figure 6B**). Flavonoid 3’-hydroxylase (F3H), chalcone synthase 1 (CHS1), and isoflavone 2’-hydroxylase (I2H) involved in flavonoid biosynthesis, as well as laccases (laccase-25 and laccase-9) involved in lignin biosynthesis were all up-regulated at 2 dpi in response to Ss97009. Simultaneously, in SsV89101 inoculated plants, a few genes involved in anthocyanin biosynthesis were induced in addition to F3H and CHS1. Cinnamoyl-CoA reductase 1 (CCR-1) and cinnamyl alcohol dehydrogenase 5 (CAD), both involved in lignin biosynthesis as well as F3H, CHS1, and anthocyanidin 3-O-glucosyltransferase were up-regulated at 5 dpi in response to Ss97009, while CHS1 and anthocyanidin reductase (2S)-flavan-3-ol-forming-like were up-regulated in SsV89101 inoculated plants at the same time point. During the sporogenesis stage, 4-coumarate-CoA ligase 3 (4-CL) and laccases (laccase-1, laccase-6 and laccase-11) were up-regulated, in response to Ss97009. Conversely, none of the genes involved in the phenylpropanoid pathway were differentially expressed in SsV89101 inoculated plants at 60 dpi. Comprehensively, Ss97009 showed a significantly higher expression of flavonoid biosynthesis genes as compared to SsV89101 during all the time periods.

ROS (Reactive oxygen species)-related responses were detected under *S. scitamineum* stress, and ROS scavenging enzymes such as glutathione S-transferase and peroxidase were differentially regulated. The majority of the glutathione S-transferase (GST) genes and peroxidase genes were up-regulated at 2 dpi in response to both Ss97009 and SsV89101. Interestingly, the induction of these genes was found to be less with Ss97009 compared to SsV89101 at 5 dpi. During the later biotrophic phase (60 dpi), Ss97009 infected plants had a few glutathione S-transferase genes up-regulated, but no genes were differentially regulated in SsV89101 inoculated plants at this time point. With regard to the differential expression of peroxidase genes at 60 dpi, most genes were down-regulated and a few were up-regulated in Ss97009 inoculated plants, while no genes were up-regulated in SsV89101 inoculated plants.

### Differential expression of genes involved in metabolic pathways

Changes in the expression level of metabolic pathways including carbohydrate metabolism were noticed in *S. scitamineum* infected plants (**Supplementary File 10**). Non-lysosomal glucosylceramidase, a sphingolipid involved in the conversion of glucosylceramide to free glucose and ceramide, was consistently up-regulated in both Ss97009 and SsV89101 inoculated plants at the initial penetration stage (2 dpi). Further, phosphoenolpyruvate carboxylase (PEPC), involved in the synthesis of oxaloacetic acid, was induced in response to both Ss97009 and SsV89101 at 5 dpi. Interestingly, at the whip emergence stage (60 dpi), cell wall invertase and vacuolar invertase, as well as trehalose-phosphate phosphatase, were all up-regulated in response to the high-virulent Ss97009, in contrast to SsV89101. In line with this, the expression level of sugar transporter SWEET4b was also found to be elevated at this time point. Additionally, endo-1,4-beta-xylanase, which hydrolyzes the xylan of cell walls, was up-regulated consistently during initial penetration (2 dpi) with both Ss97009 and SsV89101, as well as at 60 dpi in response to Ss97009.

### Resistance genes regulated during *S. scitamineum* infection

Under *S. scitamineum* stress, some of the plant resistance-related genes were differentially regulated (**Supplementary File 10**). Leucine-rich repeat protein 1 and F-box/FBD/LRR-repeat protein were up-regulated in response to both Ss97009 and SsV89101 at 2 dpi. At this stage, disease resistance protein RGA1 and leucine-rich repeat receptor-like protein kinase were down-regulated in Ss97009 and SsV89101 inoculated plants, respectively. As the colonization progresses, some of the R-genes, viz., enhanced disease resistance 4, leucine-rich repeat protein 1, and leucine-rich repeat receptor-like serine/threonine-protein kinase, were slightly up-regulated in response to Ss97009 at 5 dpi. Whereas, at this time point, R-genes, viz., probable disease resistance protein RF9 isoform X1, putative disease resistance RPP13-like protein 3, were highly elevated in SsV89101 inoculated plants. Besides, disease resistance proteins such as RPM1 and LRR receptor-like serine/threonine-protein kinase were suppressed in response to SsV89101 at 5 dpi, while no resistance-related genes were down-regulated in Ss97009 inoculated plants. Intriguingly, during the sporogenesis stage with the high-virulent Ss97009, the majority of the R-genes such as disease resistance protein Pik-2, putative disease resistance RPP13-like protein 3, leucine-rich repeat receptor-like protein kinase were down-regulated, while an LRR receptor-like serine/threonine-protein kinase was highly up-regulated in response to SsV89101.

### qPCR validation of RNA-seq data

**Figure 7:**
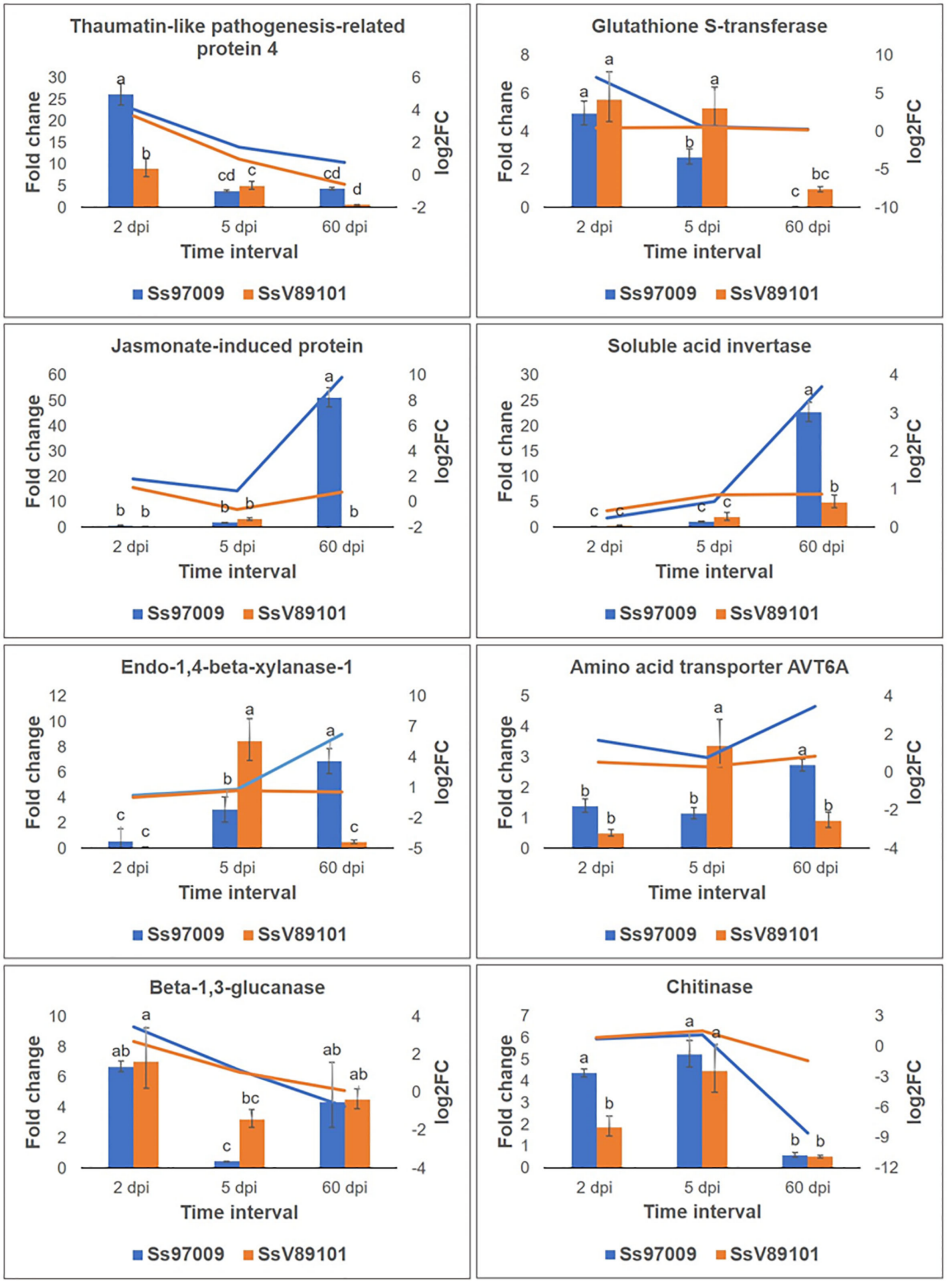
Validation of differentially expressed genes identified in Illumina sequencing by qPCR. Genes selected for validation include thaumatin-like pathogenesis-related protein 4; glutathione S-transferase; jasmonate-induced protein; soluble acid invertase; endo-1,4-beta-xylanase-1 like; amino acid transporter AVT6A; beta-1,3-glucanase; and chitinase. The x-axis represents the sugarcane buds inoculated with the *S. scitamineum* isolates Ss97009 and SsV89101 sampled at different intervals: 2 dpi, 5 dpi, and 60 dpi, the y-axis (left) represents the fold change calculated by qPCR using elongation factor α (eEF1α) gene as the reference gene, and the y-axis (right) represents the log2 fold change values obtained from Illumina sequencing. Error bars represent the standard deviation based on three biological replicates. Different small case letters over the error bars represent that they are significantly different (Tukey’s test, P≤ 0.05).

To validate the abundance of DEGs identified above, eight potential candidate DEGs, viz., thaumatin-like pathogenesis-related protein 4 (TRINITY_DN8157_c0_g1), glutathione S-transferase (TRINITY_DN17693_c0_g1), jasmonate-induced protein (TRINITY_DN986_c1_g1), soluble acid invertase (TRINITY_DN5458_c1_g2), endo-1,4-beta xylanase-1 like (TRINITY_DN25244_c0_g2), amino acid transporter AVT6A-like (TRINITY_DN3969_c0_g2), beta-1,3-glucanase (TRINITY_DN49528_c0_g1) and chitinase (TRINITY_DN95756_c0_g1) were analyzed by qPCR. Results of relative expression profiles indicated that the patterns of expression at different stages were similar to the Illumina sequencing results (**Figure 7**).

## Discussion

Plants have adapted to possess dynamic and intricate strategies to recognize and defend themselves against pathogen attack over time (Dangl and Jones, 2001; Ashwin et al., 2020b), and deciphering such defense mechanisms will be a useful strategy for developing resistant cultivars. Sugarcane is one of the most important crops in the world and any production constraint will have a profound economic impact due to its industrial potential. Its large genome size and high polyploidy level make it challenging to study and improve through genetic approaches. Hence, transcriptome sequencing is employed as a reliable tool for studying the defense mechanisms in sugarcane against biotic and abiotic stresses (Manners and Casu, 2011). Our preliminary work clearly distinguished two isolates of *S. scitamineum* as high- (Ss97009) and low-virulent (SsV89101) pathotypes, based on our phenotyping experiments performed under glasshouse conditions.

### Comparative transcriptome analysis of sugarcane infected with *S. scitamineum* isolates with different virulence patterns

In this study, comparative transcriptome analysis was used to decipher transcriptional reprogramming of the host during interaction with the pathotypes, Ss97009 and SsV89101, at the early (2 dpi and 5 dpi) and late (60 dpi) stages of the infection process. These designated time intervals gain biological significance because the initial establishment of infection critically occurs within 2 days, colonization of inter- and intracellular spaces occurs by 5 dpi, and sporogenesis followed by whip emergence occurs by 60 dpi, which were confirmed through SEM analysis and quantitative real-time PCR. The early time points, 2 and 5 dpi were crucial to understanding the differences in the initial plant defense responses (Que et al., 2014), and 60 dpi contributed to the revelation of critical molecular events that occurred during whip emergence (Schaker et al., 2016). The approach of comparative transcriptome analysis by inoculating two isolates of *S. scitamineum* differing in virulence into a single susceptible variety was attempted for the first time, resulting in a library of 6,06,157 unigenes through *de novo* transcriptome assembly. Annotation of the unigenes resulted in the identification of mostly represented organisms viz., *Sorghum bicolor*, *Zea mays*, and *Oryza sativa* related to *S. officinarum* indicating the quality of the assembled data.

Differential gene expression analysis provided vital clues on the comparative gene expression profile in sugarcane in response to inoculation with Ss97009 and SsV89101 isolates. Owing to the host interplay on limiting the fungal colonization, significant transcript changes in sugarcane were observed in response to both high- and low-virulent isolates, and the number of DEGs was larger at 2 dpi and 5 dpi compared to the whip emergence stage, presumably suggesting a strong host defense against pathogen invasion during the initial stages of disease development (Jayaswall et al., 2016). Comparatively, the number of DEGs was higher upon inoculation with Ss97009 at 2 dpi and 60 dpi, demonstrating that the host responses were more intense in response to inoculation with the high-virulent isolate than that of the low-virulent isolate. On the contrary, a slightly higher number of DEGs with SsV89101 at 5 dpi might indicate an induced defense response by a less virulent pathotype, thus leading to the suppression of disease incidence over time. The number of co-expressed genes was larger in the early stages (2 dpi and 5 dpi) than in the later stages (60 dpi), revealing that the isolates had a comparable response at the early time points. Besides, the substantial number of unique DEGs expressed at 60 dpi with the high-virulent isolate connotes that these genes might play specific roles during whip emergence.

GO annotation of DEGs revealed that the GO categories such as metabolic and biosynthetic process as well as defense-related DEGs were mostly linked to the high-virulent isolate Ss97009 suggesting the role of these genes in establishing disease development. In agreement with this, Ss97009 exhibited more COG categories participating in defense reactions than SsV89101, implying that defense responses were activated more strongly in response to the high-virulent isolate. Besides, pathway analysis demonstrated that the differentially expressed genes were related to almost all aspects of biological activities. The phenylpropanoid biosynthesis pathway was found to be outstandingly up-regulated with SsV89101 at 2 dpi, which might be a possible reason for the restriction of pathogen colonization at this early stage itself. Similarly, reprogramming of the phenylpropanoid pathway leading to increased antifungal activities was reported to be associated with resistance in soybean (Ranjan et al., 2019). Further, the down-regulation of glycolysis/gluconeogenesis was remarkably higher in SsV89101 than in Ss97009 at 5 dpi, inferring that the cells allow starvation with SsV89101. Significant down-regulation of the phenylpropanoid biosynthesis and glycerolipid metabolism pathways with Ss97009 at 60 dpi could be correlated to the biotrophic interaction between the high-virulent isolate Ss97009 and sugarcane. Overall, the functional analysis of DEGs suggests that the defense responses in response to *S. scitamineum* isolates with different virulence attributes are very complex and might be possibly regulated by multi-gene networks.

### Response of plant hormones to *S. scitamineum* infection

Plant hormones are engaged in the regulation of plant growth and development, as well as signaling networks involved in plant responses to a variety of biotic and abiotic stresses. The role of plant hormones viz., salicylic acid (SA), jasmonates (JA), and ethylene (ET) has been extensively studied and are identified as the key components of plant defense signaling (Bari and Jones, 2009). Other hormones such as auxin and gibberellic acid (GA) have also been reported to be involved in regulating the defense responses. In our study, ET and GT were the main hormones up-regulated in response to both Ss97009 and SsV89101 during the early stages of infection (2 and 5 dpi). The 1-aminocyclopropane-1-carboxylate (ACC) oxidase involved in ET biosynthesis, as well as other ethylene-responsive transcription factors induced at these time points, have been shown to play biological roles in plant developmental processes and stress responses (Licausi et al., 2013). Induction of gibberellin 20-oxidase in both Ss97009 and SsV89101 inoculated plants suggests biosynthesis of GA is associated with defense response during early stages of pathogen colonization (2 dpi and 5 dpi). Activation of JA-related genes, MYC2 transcription factor, and ZIM motif family protein, exclusively in response to Ss97009 at 2 and 5 dpi, respectively, might be an indicator of effective disease progression and high virulence. ZIM motif family protein was reported to interact with the MYC2 transcription factor, repressing its function in the JA pathway and thereby activating the expression of pathogen-responsive genes (Chini et al., 2007). Strikingly, JA-related genes were not regulated in response to the low-virulent isolate during the early stages, highlighting its crucial role in disease progression.

Differential switching of molecular responses was evidenced by the activation of a network of hormone-signaling pathways at 60 dpi in response to Ss97009, in contrast to SsV89101. Induction of auxin-related DEGs, including auxin-responsive SAUR proteins, in response to Ss97009 at 60 dpi is associated with the meristem transcriptional programming during lignin formation in the whip (Schaker et al., 2016). Besides, the changes in auxin metabolism vis-a-vis blocking apical dominance might be related to the tillering in infected plants. Up-regulation of ethylene-responsive transcription factor RAP2-6 and ACC oxidase indicate the involvement of the ET signaling pathway during whip emergence, and it is also related to the lignification of plant tissues by induction of the genes involved in the phenylpropanoid pathway (Guo and Ecker, 2004). Activation of a stress-induced 23 kDa jasmonate-induced protein was observed in response to the high-virulent isolate at 60 dpi and was reported to repress housekeeping genes including photosynthesis-related proteins (Hause et al., 1999). This might be related to the activation of pathogen-associated genes, leading to disease symptoms. The up-regulation of gibberellin 2-beta-dioxygenase involved in the deactivation of GAs, and down-regulation of gibberellin 20 oxidase 1-D involved in GA biosynthesis indicate a blockage of GA signaling during whip emission and is related to the tillering in infected plants (Lo et al., 2008). A stress-responsive mini zinc finger protein 1 up-regulated against Ss97009, which is engaged in multiple hormonal regulations was demonstrated to be associated with abnormal morphologies in *Arabidopsis* (Hu and Ma, 2006). Hence, induction of the mini zinc finger protein 1 in our study might be associated with developmental defects in the meristem during whip emission. Comprehensively, the interplay of all these genes implies that hormonal imbalance plays a key role in differential responses to distinct *S. scitamineum* isolates, which is responsible for modulating the host metabolism in their favor.

### Role of pathogenesis-related proteins

The significance of pathogenesis-related (PR) proteins in plants against pathogen attack is well established. In our study, the induction of these proteins was much more activated at 2 dpi suggesting a stronger response during initial penetration with both Ss97009 and SsV89101. Specifically, induction of typical PR proteins such as beta-1,3-glucanase and chitinase genes at 2 dpi indicates the defense against pathogen invasion. These two proteins were reported to exert higher defense responses in sugarcane during pathogen infection (Gu et al., 2008), and their antifungal activities have been demonstrated *in vitro* (Giannakis et al., 1998). Up-regulation of other PR proteins such as pathogenesis-related protein 1 and thaumatin-like proteins was comparatively higher in response to Ss97009 than SsV89101 at 5 dpi, and these proteins have also been reported to exhibit antifungal activity against many pathogens in various plant species (Lu et al., 2011; Zhang et al., 2018a). Among these, PR-1 proteins, known as the hallmark of defense pathways are the most abundant proteins induced during pathogen attack and are associated with salicylic acid-mediated disease resistance pathway (Breen et al., 2017). Another principal group of PR proteins, plant defensins, are small cysteine-rich proteins with antimicrobial activities, and the defensin-like protein P322 was up-regulated at 2 dpi in both Ss97009 and SsV89101 inoculated plants. Earlier, differential regulation of defensin genes was found to reinforce the induced defense/resistance in sugarcane against red rot (Ashwin et al., 2020a). With regard to the later biotrophic phase, the reduced number of PR proteins, as well as down-regulation of chitinase in response to the high-virulent Ss97009, could be probably linked to the susceptibility of the host, resulting in whip emergence.

### Regulation of defense-related transcription factors

In plants, transcriptional reprogramming leads to the regulation of defense responsive transcripts during pathogen invasion and is mediated by transcription factors (TFs). Majority of the TFs up-regulated in our study, viz., NAC, ERF, bHLH, MYB, and WRKY were reported to be regulated by biotic and abiotic stresses, and their potential in regulating multiple signaling networks was highlighted (Erpen et al., 2018). Among them, NAC, ERF, and bHLH35 transcription factors were up-regulated during the early stages of infection, showing that they are involved in triggering defense against pathogen invasion. Most importantly, the induction of MYB20 and WRKY12 in response to Ss97009 during the sporogenesis stage (60 dpi) reflects their role in defense responses to pathogen infection. MYB and WRKY TFs were reported to be involved in various processes, including biotic and abiotic stress responses. During secondary wall formation in *A. thaliana*, MYB20 was identified to directly activate lignin biosynthetic genes and phenylalanine biosynthetic genes (Geng et al., 2020), implying its role in lignin formation during whip emergence with the high-virulent isolate. Recently, RNA-seq analysis in a crabapple cultivar revealed that WRKY12 was closely related to the accumulation of anthocyanins, which are considered to have a variety of functions, including defense (Yang et al., 2018). In our study, WRKY TFs were not induced in response to the low-virulent isolate, confirming their vital role in mediating the whip emission. Interestingly, TFs related to meristem functions were up-regulated at 60 dpi in Ss97009 inoculated plants revealing the induction of meristem-related genes during whip emission in sugarcane. On the contrary, the less-virulent isolate had little regulation of transcription factors at 60 dpi, signifying their pivotal role in differential switching of defense signaling pathways in response to different isolates.

### Expression of genes engaged in flavonoid biosynthesis pathway

Plants produce a huge variety of metabolites, including secondary metabolites, which play key roles in defense responses against pathogens and signaling. Flavonoids are one of the major groups of metabolites and the defensive flavonoids include anthocyanidins, flavanols, isoflavonoids, etc. In our study, F3H, I2H, and CHS1, involved in flavonoid biosynthesis, were found to have a key role in defense against both the isolates during the early phases of pathogen invasion. The CHS1 involved in the flavonoid biosynthetic pathway was up-regulated at early stages (2 dpi and 5 dpi) in response to both Ss97009 and SsV89101, which were reported to be induced under *S. scitamineum* stress and in response to priming with resistance inducers in sugarcane (Que et al., 2014; Selvaraj et al., 2014). Another gene, I2H, up-regulated at 2 dpi in response to Ss97009 was shown to be involved in the biosynthesis of isoflavonoid-derived antimicrobial compounds of legumes (Akashi et al., 1998). Besides, we observed differential expression of a few genes involved in anthocyanin biosynthesis at 2 dpi, including anthocyanidin 3-O-glucosyltransferase genes, which could potentially act as antimicrobial agents, hindering invasion with the low-virulent SsV89101. Lignin deposition was another crucial event that was hampered in susceptible plants upon *S. scitamineum* infection. Here, cinnamoyl-CoA reductase 1 and cinnamyl alcohol dehydrogenase 5, involved in lignin biosynthesis, were up-regulated in response to the high-virulent isolate Ss97009 at 5 dpi. Temporary activation of these enzymes was reported in poplar infected by a necrotroph during the early stages of disease progression (Zhang et al., 2018b). Another important gene in the phenylpropanoid metabolic process, 4-coumarate-CoA ligase (4CL), was up-regulated in Ss97009 inoculated plants at 60 dpi, demonstrating its role in defense against the high-virulent isolate. Interestingly, plant laccases, which are known to lignify the secondary cell walls, were significantly up-regulated only in response to the high-virulent Ss97009 (at 2 dpi and 60 dpi). Here, the induction of laccases at 2 dpi could be associated with defense against pathogen invasion, while the induction at 60 dpi could be related to the formation of the whip, which is composed of lignified plant tissue. No flavonoid-related genes were found in response to the low-virulent SsV89101 at 60 dpi, implying that no transcriptional alterations had occurred. All these data verified that the genes involved in the flavonoid biosynthesis pathway might possibly play a role in differential switching of defense responses against different *S. scitamineum* isolates.

### Regulatory role of ROS-related genes

The association of enzymes participating in the ROS-related metabolism has been demonstrated in many plant-pathogen interactions. Here, this was evidenced by the enhanced production of ROS-related enzymes including GST and peroxidase at 2 dpi with both the isolates. The induction of these enzymes could be involved in scavenging ROS accumulated during an oxidative burst in the cell wall due to pathogen invasion, thereby reinforcing the cell wall against pathogen attack (Scheel, 1998). In our study, the host strategy to reduce the pathogen invasion by the low-virulent isolate is reflected by the higher expression of GST and peroxidase in response to SsV89101 as compared to Ss97009, at 5 dpi. Conversely, suppression of peroxidase genes by the high-virulent isolate Ss97009 at 60 dpi might presumably play a role in the compromised state of defense leading to whip emission.

### Regulation of carbon distribution in response to *S. scitamineum* infection

Apart from participating in the growth and development of the plant, primary metabolites and signaling sugars are also involved in the defense responses against various pathogens (Santa Brigida et al., 2016). Non-lysosomal glucosylceramidase, which was induced by both high- and low-virulent isolates at 2 dpi, could function as a signaling molecule in the development and pathogenicity of microbial pathogens (Wang et al., 2021). In addition, increased levels of PEPC at the early phase of infection (5 dpi) suggest the energetic demand of pathogen colonization during a compatible interaction. Previously, the PEPC was reported to be induced by a synthetic resistance inducer as well as fungal extracts at 3 and 4 dpi, contributing to defense in cucumber (Krome et al., 2007). An increase in invertase, which cleaves sucrose into glucose and fructose, has been observed in infected plant tissue during many plant-pathogen interactions (Chang et al., 2017). Here, two types of acid invertases, cell wall invertase (CWI) and vacuolar invertase (VI), were found to be up-regulated in response to Ss97009 during the whip emergence stage. The CWI is insoluble and bound to the cell wall, and plays a role in sucrose partitioning, plant development, and cell differentiation, whereas the VI is located in the vacuolar space and is involved in determining and mobilizing the sucrose level for metabolic processes (Tauzin and Giardina, 2014). Here, the up-regulation of these invertases represents a shift from sucrose storage to smut symptoms, and an increase in the hexose levels might also indicate the requirement for host nutrients (Schaker et al., 2016). Furthermore, glucose, fructose, and other sugars were also found to function as signal molecules and positively regulate the expression of defense-related genes, including PR genes (Rojas et al., 2014). Additionally, an increase in the SWEET4b gene mediating sugar transport across the plasma membrane in response to Ss97009 at 60 dpi is indicative of the active transport of sugar during the whip emergence stage, and it was shown to be induced during the invasion of fungal pathogens (Slewinski, 2011). In our study, trehalose-phosphate phosphatase 9, which removes the phosphate from trehalose 6-phosphate to produce free trehalose, was up-regulated in Ss97009 inoculated plants at 60 dpi, implying its probable role in defense responses. Trehalose, a non-reducing sugar, is a potential metabolite involved in plant-pathogen interactions, and its accumulation was found to be associated with defense responses against fungal pathogens (Renard-Merlier et al., 2007). The induction of another important gene, endo-1,4-beta-xylanase, at 2 dpi and during whip emission is supported by proteomic investigation of its differential expression during the sugarcane-*S. scitamineum* interaction (Su et al., 2016), suggesting this as one of the defense responses against the pathogen. All of these findings shed light on how key proteins involved in carbohydrate metabolism are regulated during interactions with *S. scitamineum* isolates with varying virulence characteristics.

### Response of resistance genes upon *S. scitamineum* infection

Disease resistance genes were more likely to be down-regulated during infection in susceptible plants, and the down-regulation of disease resistance protein RGA1 and leucine-rich repeat receptor-like protein kinase in response to Ss97009 and SsV89101 clearly indicates that the host resistance mechanisms were suppressed during the early phase of infection (2 dpi). RGA1 is a nucleotide-binding site leucine-rich repeat (NBS-LRR) encoding gene, and its overexpression was associated with enhanced resistance to fungal infection as well as salt and drought stresses (Li et al., 2017). Similarly, leucine-rich repeat receptor-like kinases (LRR-RLKs) were also found to be involved in regulating a wide variety of developmental and defense-related processes (Shiu et al., 2004). Intriguingly, an increase in some of the R-genes was noticed at both 2 and 5 dpi in response to these isolates, suggesting an attempt to suppress disease development during the initial penetration of bud. A higher expression of these genes in response to SsV89101 compared to Ss97009 at 5 dpi might be related to the restriction of disease development with this low-virulent isolate. At 60 dpi, several R-genes were down-regulated in whip emerged Ss97009 inoculated plants indicating the establishment of successful biotrophic interaction possibly by decreasing recognition capacity to various effectors (Jayaswall et al., 2016). Additionally, with the low-virulent isolate, the high accumulation of LRR receptor-like serine/threonine-protein kinase at 60 dpi corroborated its inefficiency in causing disease symptoms. The information on these plant disease resistance genes has increased our knowledge of resistance mechanisms, and they are potential candidates for further functional characterization, which would enhance the opportunities for generating sugarcane genotypes with durable smut resistance.

## Conclusions

**Figure 8:**
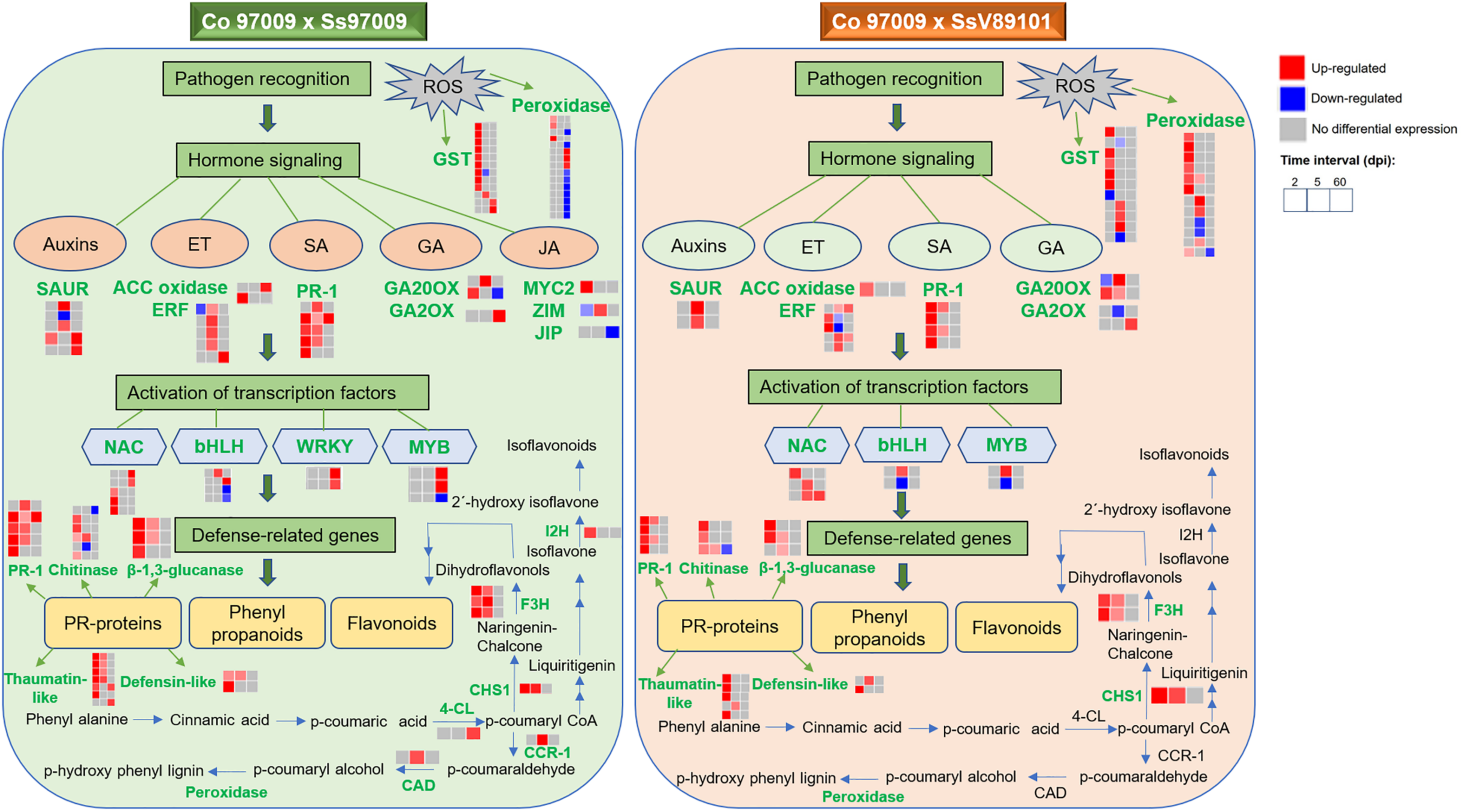
Overview of comparative gene expression profile in sugarcane cultivar Co 97009 in response to *S. scitamineum* isolates, Ss97009 (high-virulent) and SsV89101 (low-virulent). Genes shown in green are differentially expressed with the respective isolates. ET, ethylene; SA, salicylic acid; GA, gibberellic acid; JA, jasmonic acid; ACC oxidase, 1-aminocyclopropane-1-carboxylate (ACC) oxidase; ERF, ethylene-responsive transcription factor; PR-1, pathogenesis-related protein 1; GA20OX, gibberellin 20 oxidase; GA2OX, gibberellin 2-beta-dioxygenase; JIP, jasmonate-induced protein; 4-CL, 4-coumarate-CoA ligase 3; CHS1, chalcone synthase 1; F3H, flavonoid 3’-hydroxylase; I2H, isoflavone 2’-hydroxylase; CCR-1, cinnamoyl-CoA reductase 1; CAD, cinnamyl alcohol dehydrogenase; and GST, glutathione S-transferase.

In conclusion, we obtained comprehensive expression profiles of sugarcane against *S. scitamineum* isolates with varying virulence behavior at different time intervals. Our results suggest that hormone signaling, PR proteins, transcription factors, genes involved in flavonoid biosynthesis, and ROS-related genes are involved in the switching in defense responses between these two isolates with different virulence behaviors, and an integrated working model demonstrating the sequel of molecular events was summarized (**Figure 8**). Involvement of hormone signaling was evidenced by the activation of a network of hormone-related genes in response to Ss97009, indicating auxin mobilization during whip emission. Besides, the reduced number of PR proteins at 60 dpi could be convincingly attributed to the susceptibility of the host to the high-virulent Ss97009. The enhanced expression pattern of the transcription factors, including WRKY and MYB, during later stages of infection in Ss97009, was also found to implicate their significance in transcriptional reprogramming during disease development. Moreover, flavonoid biosynthesis pathway-related genes were involved in differential switching in defense responses between the high- and low-virulent isolate. With regard to ROS-related genes, suppression of peroxidase genes by the high-virulent isolate Ss97009 at 60 dpi is indicative of the compromised state of defense leading to whip emission, and the activation of carbohydrate metabolism-related genes such as invertases at this stage signifies a shift from sucrose storage to smut symptoms. Furthermore, down-regulation of R-genes is likely to be associated with the host susceptibility in whip emerged plants, which has also increased our knowledge of defense responses. Overall, this study contributed to a better understanding of the defense network in sugarcane against *S. scitamineum* and also provided information for the first time on the differential expression of genes in response to *S. scitamineum* isolates with different virulence behaviors.

## Data availability statement

The original contributions presented in the study are publicly available. This data can be found here: NCBI, PRJNA778336.

## Author contributions

NA and ARS conceived and designed the experiments. VA and RTV performed the experiments. VA and NA wrote the manuscript. NA, VA, RTV and KN are involved in analyzing the results and in revising the manuscript. ARS, PM, and RV shared their expertise in the execution of experiments and were involved in revising and improving the intellectual content of the manuscript. All authors contributed to the article and approved the submitted version.

## Funding

This work was financially supported by the Department of Biotechnology (DBT) (Sanction no. BT/PR12883/BPA/118/142/2015) and Department of Science and Technology - Science and Engineering Research Board (DST-SERB) (sanction order No. EMR/2016/006055) in the form of research grants to the corresponding author.

## Acknowledgments

The authors are grateful to The Director, ICAR-Sugarcane Breeding Institute for providing facilities and continuous encouragement. The financial support received from Department of Biotechnology (DBT), Department of Science and Technology - Science and Engineering Research Board (DST-SERB), and Indian Council of Agricultural Research (ICAR), New Delhi is greatly acknowledged. We greatly acknowledge The Director, Centre for Plant Molecular Biology and Biotechnology, and Head and other faculty of Department of Bioinformatics, Tamil Nadu Agricultural University, Coimbatore for providing the facility for NGS data analysis.

## Conflict of interest

The authors declare that the research was conducted in the absence of any commercial or financial relationships that could be construed as a potential conflict of interest.

## Publisher’s note

All claims expressed in this article are solely those of the authors and do not necessarily represent those of their affiliated organizations, or those of the publisher, the editors and the reviewers. Any product that may be evaluated in this article, or claim that may be made by its manufacturer, is not guaranteed or endorsed by the publisher.
