# Supplementary material for: Transcriptome analysis of sugarcane reveals differential switching of major defense signaling pathways in response to Sporisorium scitamineum isolates with varying virulent attributes
Source: Front Plant Sci. 2022 Oct 17;13:969826. doi: 10.3389/fpls.2022.969826 (PMC9619058; doi:10.3389/fpls.2022.969826)
Supplement: Supplementary file 1 [file DataSheet_1.pdf]

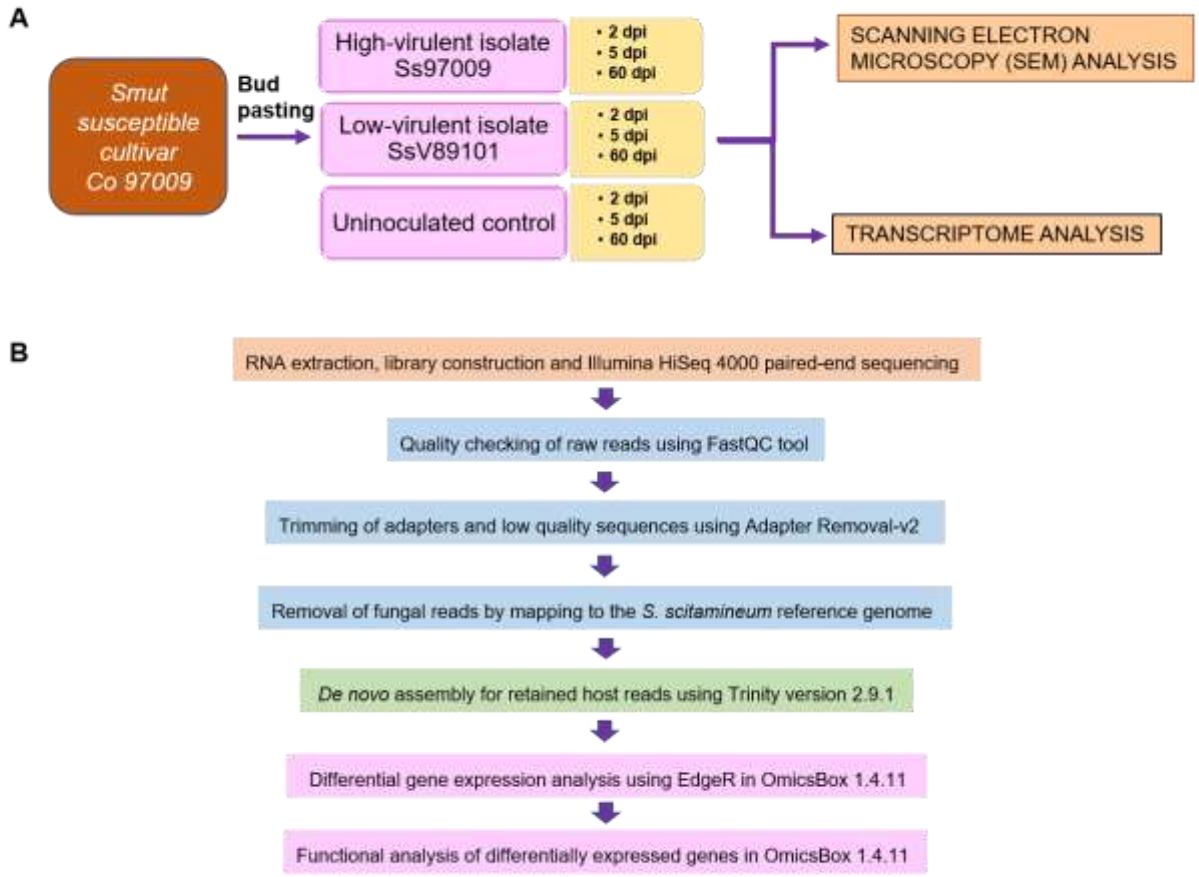

Supplementary Figure 1. Experimental design and summary of data analysis approach. **(A)** Experimental design to investigate the transcriptional changes in sugarcane against *S. scitamineum* isolates of different virulence attributes. (**B)** Workflow of Illumina sequencing and data analysis followed for dissecting the defense mechanisms in sugarcane against *S. scitaminuem* isolates.

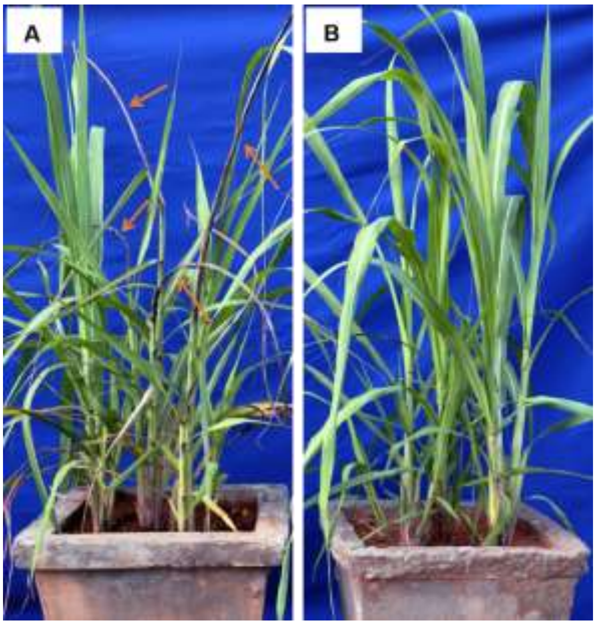

Supplementary Figure 2. Confirmation of the virulence of *Sporisorium scitamineum* isolates Ss97009 and SsV89101. **(A)** Sugarcane variety Co 97009 showing characteristic whip-shaped sori after 60 days post-inoculation (dpi) with Ss97009. **(B)** Asymptomatic plants after inoculation with SsV89101 at 60 dpi. The Co 97009 buds were inoculated with the teliospores of the respective isolates by the bud pasting method. The orange arrows indicate the whips emerging through the apical meristem.

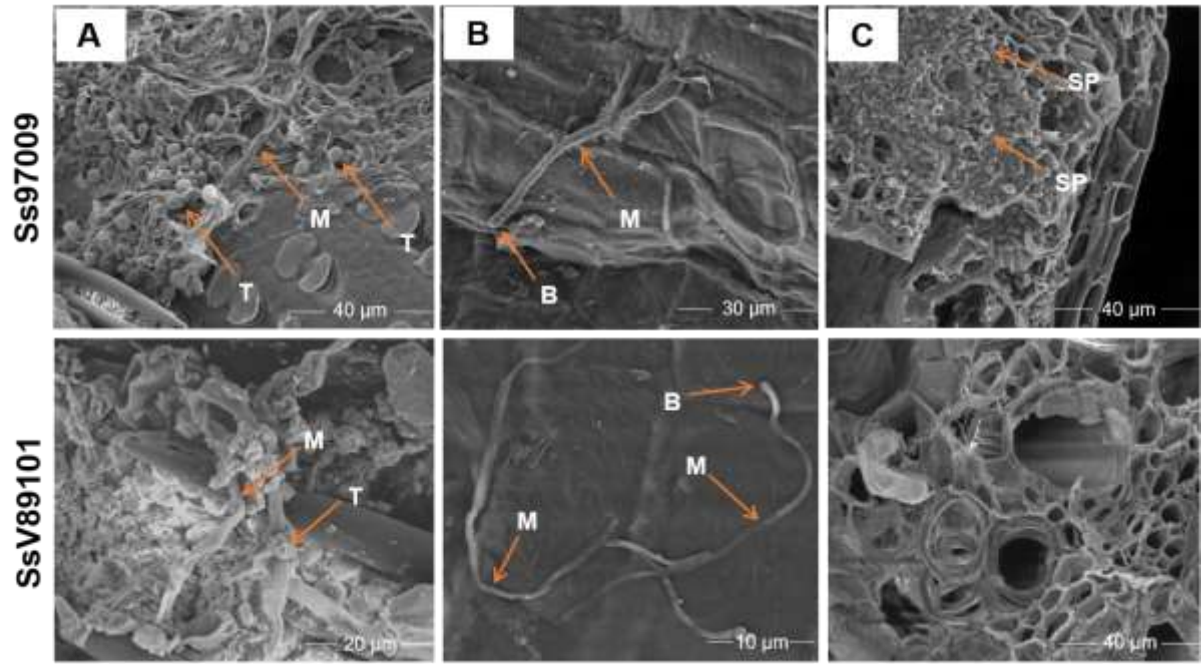

Supplementary Figure 3. *In planta* colonization stages of *S. scitamineum* isolates Ss97009 and SsV89101. **(A)** Colonization of external surface with teliospores (T), mycelia (M) with Ss97009 and SsV89101 at 2 dpi. **(B)** Mycelia (M) with bulbous structures (B) attempting penetration with Ss97009 and SsV89101 at 5 dpi. **(C)** Sporogenesis (SP) in meristem with fragmented hyphae and mass of teliospores with Ss97009, while no colonization with SsV89101 at 60 dpi.

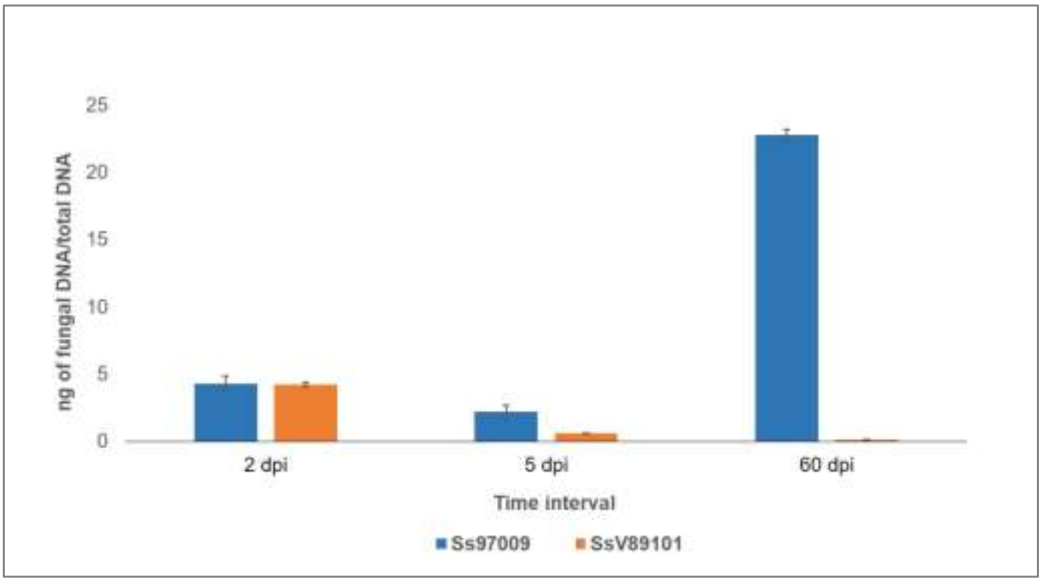

Supplementary Figure 4. Confirmation of pathogen biomass in sugarcane meristem during colonization with *S. scitamineum* isolates Ss97009 and SsV89101 by qPCR. Sugarcane cultivar Co 97009 inoculated with teliospores of the isolates Ss97009 and SsV89101 were analyzed at different time intervals, 2, 5, and 80 dpi. The results shown represent the means and standard deviations of three replicates.

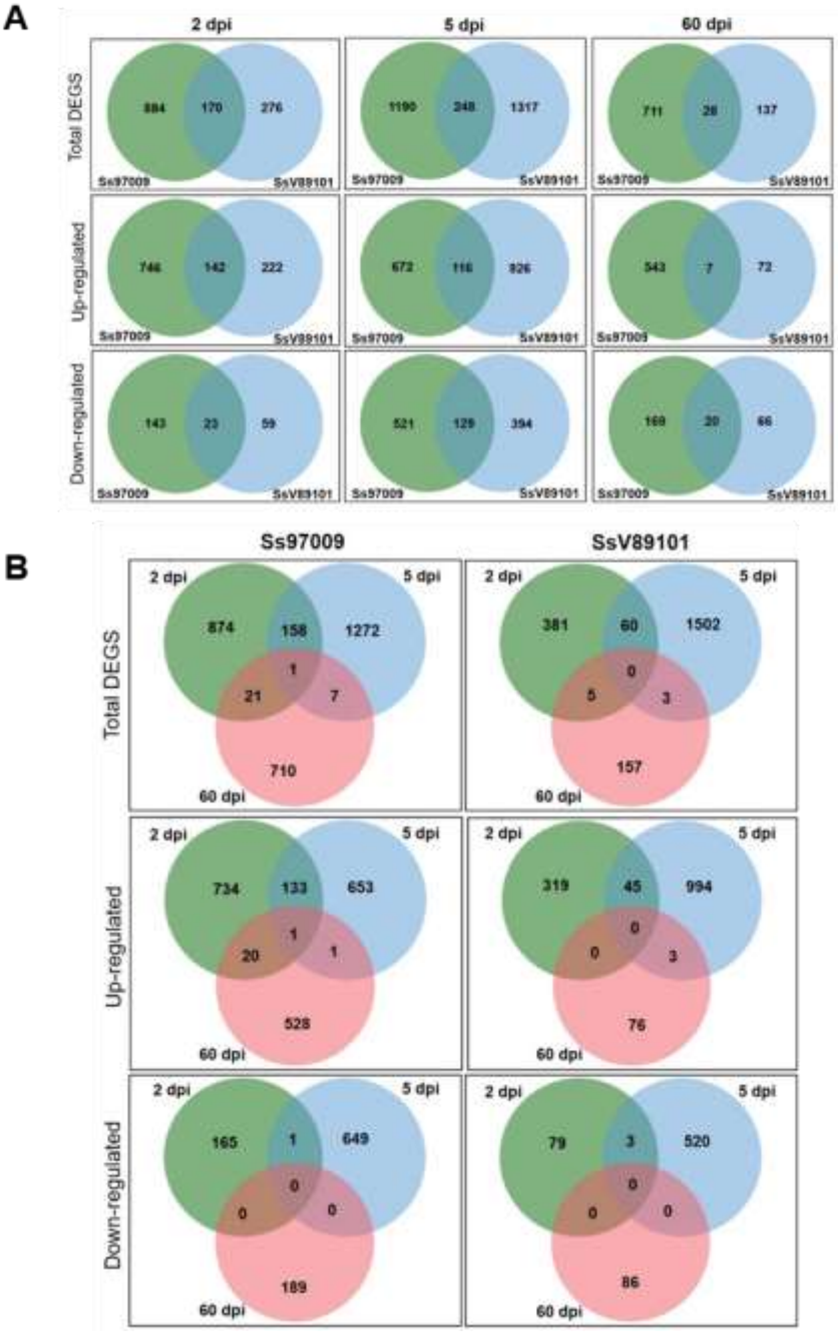

Supplementary Figure 5. Venn diagram showing differentially co-expressed genes (DEGs) in sugarcane after inoculation with the *S. scitamineum* isolates Ss97009 and SsV89101 at different time intervals. **(A)** Total, up-regulated and down-regulated DEGs between 2 dpi Ss97009 vs 2 dpi control and 2 dpi SsV89101 vs 2 dpi control; 5 dpi Ss97009 vs 5 dpi control and 5 dpi SsV89101 vs 5 dpi control; 60 dpi Ss97009 vs 60 dpi control and 60 dpi SsV89101 vs 60 dpi control. **(B)** Total, up-regulated and down-regulated DEGs between 2 dpi Ss97009 vs 2 dpi control, 5 dpi Ss97009 vs 5 dpi control and 60 dpi Ss97009 vs 60 dpi control; and DEGs between 2 dpi SsV89101 vs 2 dpi control, 5 dpi SsV89101 vs 5 dpi control and 60 dpi SsV89101 vs 60 dpi control.
