# Supplementary material for: Transcriptome analysis of sugarcane reveals differential switching of major defense signaling pathways in response to Sporisorium scitamineum isolates with varying virulent attributes
Source: Front Plant Sci. 2022 Oct 17;13:969826. doi: 10.3389/fpls.2022.969826 (PMC9619058; doi:10.3389/fpls.2022.969826)
Supplement: Supplementary file 2 [file DataSheet_2.docx]

**Supplementary Table 1.** Primers used for qPCR validation of RNA-seq analysis.

| **SI No.** | **Primer Name** | **Gene Description** | **Primer sequence** | **Amplicon size (bp)** |
| --- | --- | --- | --- | --- |
| 1 | eEF1α | Elongation factor 1α | FP: 5ʹ-GCTCTCCTTGCTTTCACCCT-3ʹ | 191 |
|  |  |  | RP: 5ʹ-TTGTCACCCTCAAAACCAGAG-3ʹ |  |
| 2 | DN8157_TLP | Thaumatin-like pathogenesis-related protein 4 | FP: 5ʹ-AAGAGCACGGGTTGATGGAC-3ʹ | 210 |
|  |  |  | RP: 5ʹ-CTTGCTCGTTGCCGAAAAGG-3ʹ |  |
| 3 | DN17693_GST | Glutathione S-transferase | FP: 5ʹ-GCTCGCTCTTGTTGCCGA-3ʹ | 162 |
|  |  |  | RP: 5ʹ-TGTGGTAGGACGGGAGAC-3ʹ |  |
| 4 | DN986_JIP | Jasmonate-induced protein | FP: 5ʹ-TCGCCCACATAGCATTCACA-3ʹ | 204 |
|  |  |  | RP: 5ʹ-CGTGCAACTTTCGAGCTTCC-3ʹ |  |
| 5 | DN5458_SAI | Soluble acid invertase | FP: 5ʹ-TGAGAGGCAAGCAAGGAAAG-3ʹ | 205 |
|  |  |  | RP: 5ʹ-ACGACTAACATGGTCACGG-3ʹ |  |
| 6 | DN25244_XYL | Endo-1,4-beta xylanase-1 like | FP: 5ʹ-TTCCAGCCCACTACAGTTGC-3ʹ | 197 |
|  |  |  | RP: 5ʹ-TCCCGGGGTGTGTAGATGAT-3ʹ |  |
| 7 | DN3969_AAT | Amino acid transporter AVT6A-like | FP: 5ʹ-GCGAGAAGTACAATGGGACG-3ʹ | 165 |
|  |  |  | RP: 5ʹ-TCCCCTTGATCAGACCATGT-3ʹ |  |
| 8 | DN49528_GLU | Beta-1,3-glucanase | FP: 5ʹ-CTCATCAACCATGTCGGGCA-3ʹ | 206 |
|  |  |  | RP: 5ʹ-GTATATATGGACCCGGCGAC-3ʹ |  |
| 9 | DN95756-CHI | Chitinase | FP: 5ʹ-GGCGACAACTTGGACTGCTA-3ʹ | 183 |
|  |  |  | RP: 5ʹ-TGCATACGCTCTGACGTCTC-3ʹ |  |

**Supplementary Table 2.** Summary of the RNA-seq raw data and quality assessment of sugarcane transcriptome.

| **Sample Name** | **No. of raw reads (PE)** | **No. of bases (GB)** | **GC (%)** | **% data >=Q30** | **Raw read length (bp)** |
| --- | --- | --- | --- | --- | --- |
| 2dpi Control-R1 | 2,47,98,816 | 7.44 | 51.73 | 96.2 | 150 x 2 |
| 2dpi Control-R2 | 2,12,97,491 | 6.39 | 52.12 | 96.1 | 150 x 2 |
| 2dpi Control-R3 | 2,23,76,716 | 6.71 | 52.25 | 96.1 | 150 x 2 |
| 2dpi Ss97009-R1 | 2,92,52,873 | 8.78 | 52.94 | 96 | 150 x 2 |
| 2dpi Ss97009-R2 | 2,55,37,838 | 7.66 | 52.95 | 95.9 | 150 x 2 |
| 2dpi Ss97009-R3 | 2,71,01,561 | 8.13 | 52.76 | 96 | 150 x 2 |
| 2dpi SsV89101-R1 | 2,47,99,652 | 7.44 | 52.84 | 96.2 | 150 x 2 |
| 2dpi SsV89101-R2 | 3,39,11,995 | 10.17 | 51.36 | 95.8 | 150 x 2 |
| 2dpi SsV89101-R3 | 2,90,68,939 | 8.72 | 50.86 | 95.8 | 150 x 2 |
| 5dpi Control-R1 | 1,86,57,039 | 5.6 | 50.65 | 95.2 | 150 x 2 |
| 5dpi Control-R2 | 1,93,99,032 | 5.82 | 49.92 | 95.9 | 150 x 2 |
| 5dpi Control-R3 | 1,69,83,782 | 5.09 | 48.99 | 94.1 | 150 x 2 |
| 5dpi Ss97009-R1 | 2,63,19,304 | 7.9 | 51.37 | 95.8 | 150 x 2 |
| 5dpi Ss97009-R2 | 2,37,87,408 | 7.14 | 50.95 | 96 | 150 x 2 |
| 5dpi Ss97009-R3 | 2,41,54,757 | 7.25 | 50.73 | 96.1 | 150 x 2 |
| 5dpi SsV89101-R1 | 2,08,37,587 | 6.25 | 48.94 | 96.1 | 150 x 2 |
| 5dpi SsV89101-R2 | 1,80,56,653 | 5.42 | 48.84 | 96 | 150 x 2 |
| 5dpi SsV89101-R3 | 2,19,59,118 | 6.59 | 50.15 | 95.7 | 150 x 2 |
| 60dpi Control-R1 | 2,49,78,809 | 7.49 | 49.44 | 95.9 | 150 x 2 |
| 60dpi Control-R2 | 2,88,59,211 | 8.66 | 49.43 | 95.9 | 150 x 2 |
| 60dpi Control-R3 | 3,34,02,298 | 10.02 | 50.04 | 96.1 | 150 x 2 |
| 60dpi Ss97009-R1 | 2,35,45,133 | 7.06 | 50.33 | 95.6 | 150 x 2 |
| 60dpi Ss97009-R2 | 1,88,35,585 | 5.65 | 50.17 | 95.8 | 150 x 2 |
| 60dpi Ss97009-R3 | 2,15,28,324 | 6.46 | 50.22 | 95.7 | 150 x 2 |
| 60dpi SsV89101-R1 | 2,27,40,707 | 6.82 | 49.12 | 95.7 | 150 x 2 |
| 60dpi SsV89101-R2 | 2,29,09,898 | 6.87 | 48.34 | 95.9 | 150 x 2 |
| 60dpi SsV89101-R3 | 2,86,68,379 | 8.6 | 49.48 | 95.8 | 150 x 2 |

*The sample names represent the sugarcane cultivar Co 97009 inoculated with the S. scitamineum isolates Ss97009 and SsV89101 at different time intervals (2 dpi, 5 dpi, and 60 dpi). Mock inoculated control samples were sampled at the same intervals, and R1, R2, and R3 indicate the biological replicate number.*

**Supplementary Table 3.** Summary of the alignment data set considered for assembly.

| **Sample Name** | **Total Read Count** | **Read count after rRNA removal** | **QC Pass %** | **Aligned Read Count with Ss*** | **Aligned reads with Ss* (%)** | **Unaligned reads with Ss* (%)** |
| --- | --- | --- | --- | --- | --- | --- |
| 2dpi-Control-R1 | 4,95,97,632 | 3,25,97,990 | 65.72 | 20,138 | 0.06 | 99.94 |
| 2dpi-Control-R2 | 4,25,94,982 | 2,43,58,884 | 57.19 | 12,012 | 0.05 | 99.95 |
| 2dpi-Control-R3 | 4,47,53,432 | 2,53,31,080 | 56.6 | 12,000 | 0.05 | 99.95 |
| 2dpi-Ss97009-R1 | 5,85,05,746 | 3,20,69,844 | 54.81 | 46,740 | 0.15 | 99.85 |
| 2dpi-Ss97009-R2 | 5,10,75,676 | 2,93,64,428 | 57.49 | 37,248 | 0.13 | 99.87 |
| 2dpi-Ss97009-R3 | 5,42,03,122 | 3,14,49,914 | 58.02 | 1,48,904 | 0.47 | 99.53 |
| 2dpi-SsV89101-R1 | 4,95,99,304 | 2,90,16,084 | 58.5 | 71,040 | 0.24 | 99.76 |
| 2dpi-SsV89101-R2 | 6,78,23,990 | 5,70,05,758 | 84.05 | 70,976 | 0.12 | 99.88 |
| 2dpi-SsV89101-R3 | 5,81,37,878 | 4,47,65,542 | 77 | 69,266 | 0.15 | 99.85 |
| 5dpi-Control-R1 | 3,73,14,078 | 2,87,33,886 | 77.01 | 10,120 | 0.04 | 99.96 |
| 5dpi-Control-R2 | 3,87,98,064 | 3,64,34,130 | 93.91 | 14,778 | 0.04 | 99.96 |
| 5dpi-Control-R3 | 3,39,67,564 | 2,71,25,060 | 79.86 | 5,91,490 | 2.18 | 97.82 |
| 5dpi-Ss97009-R1 | 5,26,38,608 | 4,02,22,290 | 76.41 | 26,132 | 0.06 | 99.94 |
| 5dpi-Ss97009-R2 | 4,75,74,816 | 3,64,73,444 | 76.67 | 22,254 | 0.06 | 99.94 |
| 5dpi-Ss97009-R3 | 4,83,09,514 | 3,79,99,482 | 78.66 | 20,964 | 0.06 | 99.94 |
| 5dpi-SsV89101-R1 | 4,16,75,174 | 3,67,19,820 | 88.11 | 60,470 | 0.16 | 99.84 |
| 5dpi-SsV89101-R2 | 3,61,13,306 | 3,26,18,146 | 90.32 | 72,942 | 0.22 | 99.78 |
| 5dpi-SsV89101-R3 | 4,39,18,236 | 3,67,89,482 | 83.77 | 53,456 | 0.15 | 99.85 |
| 60dpi-Control-R1 | 4,99,57,618 | 3,96,21,188 | 79.31 | 15,784 | 0.04 | 99.96 |
| 60dpi-Control-R2 | 5,77,18,422 | 4,77,98,110 | 82.81 | 39,556 | 0.08 | 99.92 |
| 60dpi-Control-R3 | 6,68,04,596 | 5,44,59,570 | 81.52 | 17,980 | 0.03 | 99.97 |
| 60dpi-Ss97009-R1 | 4,70,90,266 | 4,23,15,166 | 89.86 | 1,04,08,870 | 24.6 | 75.4 |
| 60dpi-Ss97009-R2 | 4,54,81,414 | 4,44,92,714 | 97.83 | 1,10,07,238 | 24.74 | 75.26 |
| 60dpi-Ss97009-R3 | 4,30,56,648 | 4,00,02,472 | 92.91 | 99,78,668 | 24.95 | 75.05 |
| 60dpi-SsV89101-R1 | 3,76,71,170 | 3,04,99,916 | 80.96 | 12,186 | 0.04 | 99.96 |
| 60dpi-SsV89101-R2 | 4,58,19,796 | 4,27,07,456 | 93.21 | 19,312 | 0.05 | 99.95 |
| 60dpi-SsV89101-R3 | 5,73,36,758 | 4,34,75,294 | 75.82 | 18,062 | 0.04 | 99.96 |

Ss* - *Sporisorium scitamineum* reference genome

**Supplementary Table 4.** *De novo* assembly results of sugarcane transcriptome using Trinity software.

| **Description** | **Trinity unigenes** | **Trinity transcript (isoforms)** |
| --- | --- | --- |
| Total counts | 6,06,157 | 11,65,148 |
| Longest transcript length (bp) | 48,175 | 23,789 |
| Mean GC% | 46.66 | 46.49 |
| Contig N10 | 3,250 | 4,332 |
| Contig N20 | 2,011 | 2,538 |
| Contig N30 | 1,267 | 1,519 |
| Contig N40 | 833 | 989 |
| Contig N50 | 584 | 689 |
| Median contig length | 312 | 335 |
| Average contig length | 505.62 | 562.50 |
| Total assembled bases | 246,313,827 | 340,966,147 |
